# Depth profiles of the interfacial strains of Si_0.7_Ge_0.3_/Si using three-beam Bragg-surface diffraction

**DOI:** 10.1038/srep25580

**Published:** 2016-05-09

**Authors:** Yan-Zong Zheng, Yun-Liang Soo, Shih-Lin Chang

**Affiliations:** 1 Department of Physics, National Tsing Hua University, 101, Section 2, Kuang Fu Road, Hsinchu, Taiwan 30013; 2 National Synchrotron Radiation Research Center, 101, Hsin-Ann Road, Hsinchu Science Park, Hsinchu, Taiwan 30076.

## Abstract

Interfacial strains are important factors affecting the structural and physical properties of crystalline multilayers and heterojunctions, and the performance of the devices made of multilayers used, for example, in nanowires, optoelectronic components, and many other applications. Currently existing strain measurement methods, such as grazing incidence X-ray diffraction (GIXD), cross-section transmission electron microscope, TEM, and coherent diffractive imaging, CDI, are limited by either the nanometer spatial resolution, penetration depth, or a destructive nature. Here we report a new non-destructive method of direct mapping the interfacial strain of [001] Si_0.7_Ge_0.3_/Si along the depth up to ~287 nm below the interface using three-beam Bragg-surface X-ray diffraction (BSD), where one wide-angle symmetric Bragg reflection and a surface reflection are simultaneously involved. Our method combining with the dynamical diffraction theory simulation can uniquely provide unit cell dimensions layer by layer, and is applicable to thicker samples.

Bragg-surface diffraction (BSD)^1^,^2^,^3^,^4^ occurs when the sample crystal is first aligned for a symmetric Bragg reflection, say G, by adjusting the Bragg angle *θ*_*B*_ and then the crystal is rotated by the azimuth, ϕ, around the reciprocal lattice vector 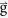 of the G-reflection, without disturbing the G reflection, to bring an additional surface reflection, L, also satisfying Bragg’s law (Fig. 1a). In reciprocal space (Fig. 1b), three reciprocal lattice points (r.l.p.), O, G, and L, are moved onto the surface of the Ewald sphere. Thus three r.l.p., O, G, and L, lie simultaneously on the surface of the Ewald sphere and excite three beams along the wavevectors, 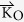, 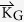 and 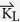. Three-beam BSD thus occurs^5^. For clarity, (G/L) is used to denote the three-beam BSD. Since point L is on the equatorial plane, the reflected beam is propagating along the crystal surface, thus the grazing-exit diffraction, L, provides scattering information from the surface, interface, up to the depth comparable with the extreme depth limitation by the evanescent waves of the Bragg reflection G. The geometries of the real and reciprocal space are shown in Fig. 1a,b, respectively.

The heterojunction^6^,^7^,^8^,^9^, Si_0.7_Ge_0.3_/Si, is prepared by using a germanium silicide target to grow a thin-film of Si_0.7_Ge_0.3_ on silicon substrate by molecular beam epitaxy, MBE, in National Nano Device Laboratory, NDL. In addition, before the epitaxial process, the Si wafer clearance is a standard manufacturing procedure, i.e., the surface particles and native SiO_2_ are removed. An energy dispersive spectrum, EDS, is employed to measure compositions versus depth (Si ~0.7 ± 0.01 and Ge ~0.3 ± 0.01, see Fig. 2d). The θ–2θ scans, namely the q-scans, of the conventional X-ray Bragg diffractions (shown in Fig. 2a–c) from 400, 040 and 004 reflections at 12 keV reveal that the a- and b-axis of the two materials, Si_0.7_Ge_0.3_ and silicon, are very close to each other. By the modelling data (solid line in red) of Fig. 2a–c, the a- and b-axis are about 5.428 and 5.4256 Å, respectively, in the Si_0.7_Ge_0.3_ thickness, d_SiGe_ (~57 nm). The c-axes of the Si_0.7_Ge_0.3_ and silicon substrate are about 5.5344 and 5.4345 Å, because of two distinct 004 peaks (Fig. 2c). In such an epitaxial process, the most stable heterostructure occurs when the a- and b-axis of the Si_0.7_Ge_0.3_ are compressed to match that of the Si substrate, along with the stretched c-axis of the Si_0.7_Ge_0.3_. Here, referring to the measured bulk value, 5.4898 Å, of Si_0.7_Ge_0.3_, the tensile strains along the a-, b- and c-axis are about −0.011, −0.011 and 0.008, respectively. Furthermore, the epitaxial process not only caused the tensile strains but also shear strains, thus the lattice parameters under study are assumed to have the degrees of freedom along the tensile and shear directions. To confirm the lateral distribution of the thin-film system, the X-ray reflectivity, XRR, is adopted to measure the thickness of Si_0.7_Ge_0.3_ and the roughnesses of the surface and interface of the Si_0.7_Ge_0.3_/Si. The experiment and simulation of the XRR data (see ref. 10) are shown in Fig. 2e. From the simulation, the Si_0.7_Ge_0.3_ thickness, *d*_SiGe_, is about 58.5 nm. The roughnesses of the surface (*σ*_surface_) and interface (*σ*_interface_) are about 0.9 and 1.1 nm, respectively. Moreover, through the image of high-resolution transmission electron microscope, HRTEM, the local probe of the Si_0.7_Ge_0.3_ thickness is about 59.9 nm (Fig. 2f), and the interfacial lattice array is also shown in Fig. 2g.

Due to the structural proximity of the Si_0.7_Ge_0.3_ film and the Si substrate, three sets of three-beam BSD, 004/202, 004/0
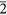
2 and 004/4
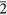
2, for Si_0.7_Ge_0.3_ and three surface reflections, 202, 0
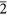
2 and 4
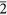
2, for Si substrate are measured along the vertical two theta (tth) and horizontal (beta) scans of the surface diffracted beams (Fig. 2a). The 004 is a wide-angle symmetric reflection, which ensures large penetration in the crystal due to the large incident angle. The three lattice unit vectors, 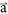, 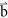 and 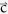 of Si_0.7_Ge_0.3_ and Si unit cells are estimated by matching the measured and the calculated intensity distributions, for both tth- and beta-scans. The combined boundary conditions of EM fields, covering each atomic layer along [001], are considered in the calculations based on the dynamical theory of X-ray diffraction for layered crystalline material^11^,^12^. For an X-ray penetration depth about 287 nm (see Supplementary Figure 5), 1000 atomic layers of about 5.43 Å thick each are considered. Since the same 004 reflection is involved in the three sets BSD’s, the three lattice unit vectors can be uniquely determined along the depth^13^.

The BSD diffraction experiments of Si_0.7_Ge_0.3_/Si are carried out at beamline 17B1, Taiwan Light Source (TLS), National Synchrotron Radiation Research Center (NSRRC). For the BSD, 004/202, the sample is mounted at the center of an 8-circle diffractometer. For 12 keV X-rays, after the alignment for the symmetric Bragg reflection, 004, and the surface reflection, 202, the intensity distributions of vertical (tth) and horizontal (beta) scans of the surface 202 reflection are measured with the slit sizes 10 mm(H) × 0.1 mm(V) and 0.1 mm(H) × 0.1 mm(V), respectively.

Figure 3a shows schematically the tth- and beta-scans of the 202 surface diffraction. For the tth-scan, the two peaks p1 and p4 are the 202 surface diffractions from Si_0.7_Ge_0.3_ and Si respectively. The other two peaks, p2, p3 and the oscillating background are the Kiessig fringes^14^ due to the 202 surface diffraction. Both the measured and the simulated profiles of the tth-scan of 202 are shown in Fig. 3b, where the simulation is based on the dynamical theory^3^,^11^,^12^,^15^. The corresponding beta-scans at p1, p4, p2 and p3 are shown in Fig. 2c–f, respectively. All the experimental data are plotted in square dot and the simulated curves are depicted with solid line.

The tth-scan of surface diffraction, 0
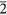
2 in the BSD, 004/0
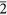
2, is shown in Fig. 4a. Figure 4b–e show the beta-scans at p1, p4 and p2, p3 of Fig. 4a, respectively. Similarly, the surface diffraction, 4
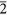
2 of the BSD, 004/4
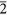
2, are in Supplementary Figure 8.

The simulations are started from finding unique solutions for the three lattice unit vectors, 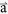, 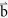 and 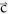, of the Si_0.7_Ge_0.3_ thin-film and silicon substrate so that the simulated intensity distributions fit the measured ones. Namely, the elements of the metric tensor, *σ*, defined below will be determined,


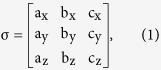


where 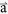 = (a_x_, a_y_, a_z_), 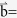 (b_x_, b_y_, b_z_) and 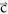 = (c_x_, c_y_, c_z_). The peak positions of the tth- and beta- scans of the three surface reflections, 202, 0
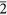
2 and 4
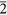
2 given above are used to solve the tensor *σ*.

According to Supplement Ι, the measured tth and beta of p1 from the three surface diffractions (Figs 3b–f and 4 and Supplementary Figure 8) give the nine elements of the Si_0.7_Ge_0.3_, denoted as *σ*_1_. Similarly, the measured tth and beta of p4 from Figs 2 and 3 determine the nine elements of the Si substrate as *σ*_2_. For clarity, the tensor elements of *σ* are labeled as (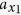, 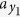, 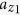), (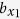, 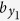, 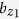), (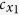, 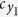, 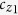) and that of *σ*_2_ as (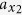, 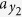, 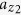), (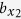, 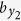, 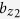), (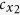, 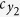, 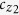).

As the samples are high-quality crystals with narrow peak widths (Fig. 2c), the dynamical theory of X-ray diffraction^3^,^11^,^12^,^15^ is employed in the simulations. The interface of Si_0.7_Ge_0.3_/Si is usually very sharp, however, in terms of an Angstrom scale, the interface can be treated as a gradual change from the Si_0.7_Ge_0.3_ thin-film to silicon substrate. Furthermore, with the EDS data (Fig. 2e) and Vegard’s law, the predictions of the lattice parameter vs. depth follow approximately the Boltzmann function (see Supplementary Figure 4). Namely, the nine elements of the tensor *σ* for each atomic layer can be determined from the *σ*_1_ (Si_0.7_Ge_0.3_) and *σ*_2_ (Si) at corresponding depth, x, using the Boltzmann function as an approximation:


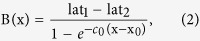


where the symbol, x, is the depth from the crystal surface, and x_0_ is the thickness of the Si_0.7_Ge_0.3,_ determined from the Bragg peak at p1. The two constants, lat_1_ and lat_1_, are the corresponding elements of the tensors, *σ*_1_ and *σ*_2_, respectively. For example, for Β(x) at depth, x, 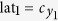 and 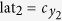. The symbol *c*_0_ is the changing rate of lattice parameters in depth, which is determined by fitting the simulations to measured intensity distributions.

To determine the changing rate *c*_0_ of the lattice parameters for the Si_0.7_Ge_0.3_/Si sample of a thousand atomic layers along [001], the multilayer dynamical theory of X-ray diffraction^12^ is employed to deal with multiple boundaries. The simulations start with Maxwell’s equations, whose solutions as Bloch functions leading to the Fundamental equations of wavefield, Supplementary equation (10) (See Supplement II) which can be solved as an eigenvalue equation by using the formalism in single Cartesian coordinates^11^. 4N modes with 4N eigenvectors and eigenvalues are involved in an N-beam diffraction with N ≥ 2 (see Supplement III) for each atomic layer. By applying the boundary conditions, the continuities of the tangential components of the electric field, 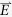 and magnetic field, 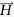, and the normal components of the electric displacement, 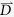 and magnetic induction, 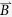 (Supplement IV), the diffracted E fields at the *n*^th^ atomic layer are calculated as 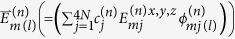, which is the sum of the 4N E-fields (eigenvectors) with the 4N proportional coefficients 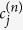 and their corresponding phases, 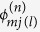, at the top boundary (*l* = 1) and bottom boundary (*l* = 2) of the *n*^th^ layer considered. For multilayers, the coefficients, 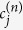 are solved by the boundary conditions for the top (entrance) surface, the intermediate boundary from *n*^th^-to-(*n* + 1)^th^ layers, and the bottom (exit) surface of the sample. This leads to the diffracted E-fields given in Supplementary equation (33) (see, Supplement V). For each surface reflection the calculations involve 1000 atomic layers and 4N modes (N = 3 for three beam BSD) for each layer. Therefore a linear system of 4 × 3 × 1000 equations are simultaneously solved to give the calculated profiles (tth- and beta-scans) of the three surface reflections, 202, 0
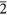
2 and 4
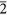
2, respectively.

Moreover, due to the shallow diffraction angles of the surface reflections with respect to the crystal surface, part of the diffracted beams are absorbed by the crystal. Therefore a fitting function 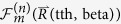 at the position vector, 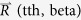, of the detector from the sample is given to each surface reflection, where the diffraction geometry, acceptance angle of the detector, beam divergence, and the miscut angle between the surface and atomic layers are considered (See, Supplement VI). The fitting functions are plotted in Supplement VII. The interference among the diffracted beams inside the crystal is also included. This leads to the diffracted intensity, *I*_*m*_, as below:


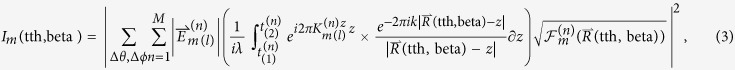


where 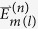 is the diffracted E-field of the *n*^*th*^ layer’s top boundary for the *m*^th^ reflection. Note that m = 0 is for the forward diffracted beam O, *m* = 1 for the symmetric Bragg reflection G, and *m* = 2 for the surface diffraction L, and 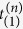 and 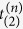 are the vertical positions of the top and bottom boundaries of the *n*^*th*^ layer. 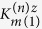 is the z-component of the wavevector 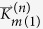 outside the crystal of the *n*^th^ layer’s top boundary for the *m*^th^ reflection. The terms, Δ*θ* (~0.007 deg.) and Δ*ϕ* (~0.02 deg.) are the vertical and horizontal divergences, respectively on the beamline BL17B1. The symbol, M is the total number of atomic layers of the simulation i.e, M = 1000. The position vector, 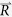, is shown in Supplementary equation (35).

The best fit between the simulated intensity distributions to the measured ones is reached when *c*_0_ = 0.6 for these cases. When *c*_0_ < 0.6, the measured intensity distributions of the interference between the diffraction from the Si_0.7_Ge_0.3_ and from the Si substrate cannot be matched by the simulated curves. When *c*_0_ > 0.6, the simulated peak intensities at p1 and p4 will be off the measured values. With *c*_0_ = 0.6, the nine elements of the σ tensor as functions of depth in Å are determined, which are shown in Fig. 5a–d. The coresponding strains, Fig. 5e–h, are estimated with the expected ones which are predicted from the EDS data, Fig. 2d, and Vegard’s law (See, Supplement IX). The uniqueness of lattice parameter determination is also verified (See, Supplement X), such that the determined lattice parameters fit all the BSD measurements simultaneously.

Furthermore, the six lattice parameters can be also calculated from the σ-tensor. The results are shown in Fig. 6a[Fig f6]b and the blow-ups of the interface in Fig. 6c,d. After the thin film deposition, the parameter α is slightly larger and β is slightly smaller than 90 degrees indicating that the c-axis deviates from the normal direction, [001], due to the thin-film growth so that the two angle, α and β are not exactly equal to 90° (α ≥ 90° and β ≤ 90° in all depths). Due to the growth direction perpendicular to the [100] or [010], the variation of the angle, γ, is the smallest. When the thin-film, Si_0.7_Ge_0.3_, are grown on the silicon wafer, the a- and b-axis of the thin-film, Si_0.7_Ge_0.3_, are bound to the silicon substrate. To relax the strain, the c-axis of the thin-film, Si_0.7_Ge_0.3_, is longer than a- and b-axis, and the structure became rectangular rather than cubic^16^. Also, the silicon substrate inside the probing limit (~300 nm) is slightly distorted as a triclinic structure, and its c-axis is stray from the direction, [001] due to the thin-film deposition.

In conclusion, the depth profiles of interfacial strains and lattice parameters of Si_0.7_Ge_0.3_/Si are successfully obtained. This method can be used for strain mapping of a wide variety of multilayer crystal systems (For imperfect multilayers, the simulations can be replaced by the power-transfer equation of the kinematical theory^17^), which is useful for polarization engineering in ferroelectricity^18^ and nanotechnology applications^19^,^20^,^21^,^22^,^23^,^24^,^25^. Especially, the shear and tensile strains may be applied to offer sufficient information to control the interfacial carrier mobility in the so-called stain-engineering^26^,^27^,^28^,^29^,^30^ for devices. With the advent of new X-ray sources beyond diffraction-limit, the BSD of better coherence may improve the strain resolution further.

## Additional Information

**How to cite this article**: Zheng, Y.-Z. *et al*. Depth profiles of the interfacial strains of Si_0.7_Ge_0.3_/Si using three-beam Bragg-surface diffraction. *Sci. Rep.* **6**, 25580; doi: 10.1038/srep25580 (2016).

## Supplementary Material

Supplementary Information

## Figures and Tables

**Figure 1:**
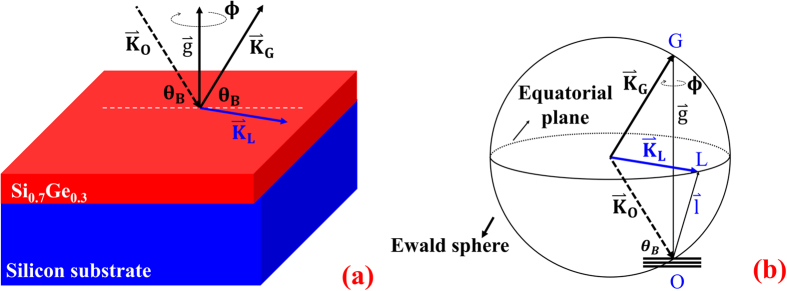
BSD’s diffraction geometry . (**a**) The schematic representation of there-beam Bragg-Surface diffraction from the bilayer Si_0.7_Ge_0.3_ (in red)/Si (in blue) in real space: the forward diffracted beam with the wavevector, 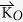, is reflected by the sample at the Bragg angle, θ_B_, to generate the symmetric reflected beam with the wavevector, 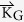. The azimuth rotation, ϕ, around the reciprocal lattice vector, 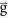, of the symmetric reflection, G, then excites the surface reflection along 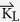, which is parallel to the surface. (**b**) The BSD in reciprocal space: the ϕ rotation brings an additional reciprocal lattice point, L, onto the surface of the Ewald sphere in the equatorial plane, thus three reciprocal lattice points, O, G, L being on the surface of the Ewald sphere simultaneously.

**Figure 2:**
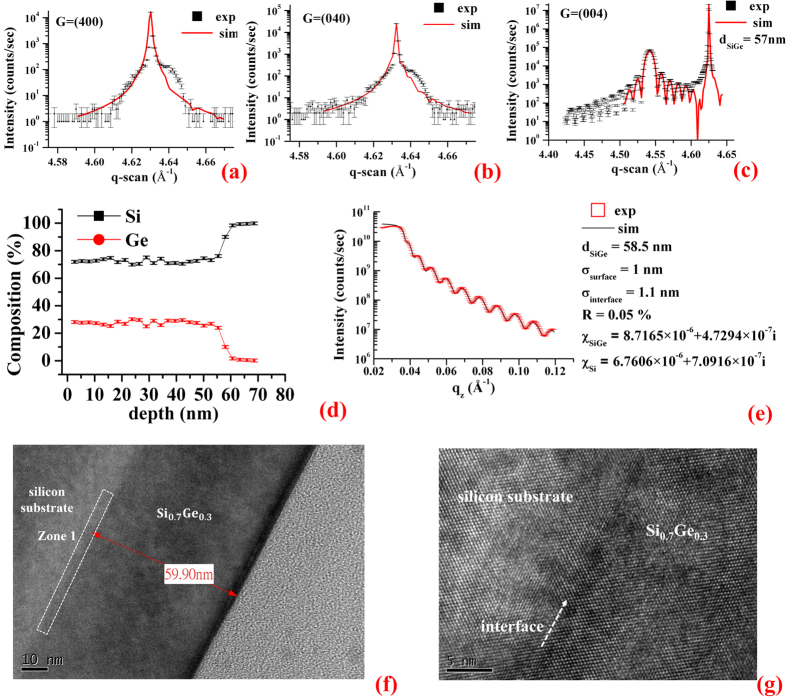
Material characterization. (**a**) The experiments (square in black) and fitting curves (solid line in red) show the q-scans of 400, (**b**) 040, and (**c**) 004. The fitting curves are simulated by the SiGe thickness, d_SiGe_ (~57 nm). In the q-scan of the 004 reflection, the sharp peak is due to Si substrate and the broad peak is from Si_0.7_Ge_0.3_ thin film. Interference fringes due to two layer system are also observed. (**d**) Energy Dispersive Spectrum, EDS, shows that the compositions of silicon and germanium about 0.7 and 0.3, respectively, distributed from the top (0 nm) to 57 ± 4 nm in depth (the analysis are in Supplementary Figure 7). (**e**) The experiment (open square in red) and simulation (solid line in black) of the X-ray reflectivity, XRR, measurements give the SiGe thickness (*d*_SiGe_ ~ 58.5 nm), surface roughness (*σ*_surface_ ~ 1 nm) and interface roughness (*σ*_interface_ ~ 1.1 nm) according to the refractive index, 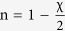 (where χ is the dielectric susceptibility). The obtained dielectric susceptibilities of SiGe and Si are χ_SiGe_(~8.7165 × 10^−6^ + 4.7294 × 10^−4^i) and χ_Si_(~6.7606 × 10^−6^ + 7.0916 × 10^−4^i) with the reliability, R(~0.05%), of the curve-fitting. (**f**) High-resolution Transmission Electron Micrograph shows the lateral image of the hetero structure, Si_0.7_Ge_0.3_/Si, and reveals its thickness being about 59.9 nm. (**g**) Interface of the hetero structure, Si_0.7_Ge_0.3_/Si, is located in Zone 1 of (**f**).

**Figure 3:**
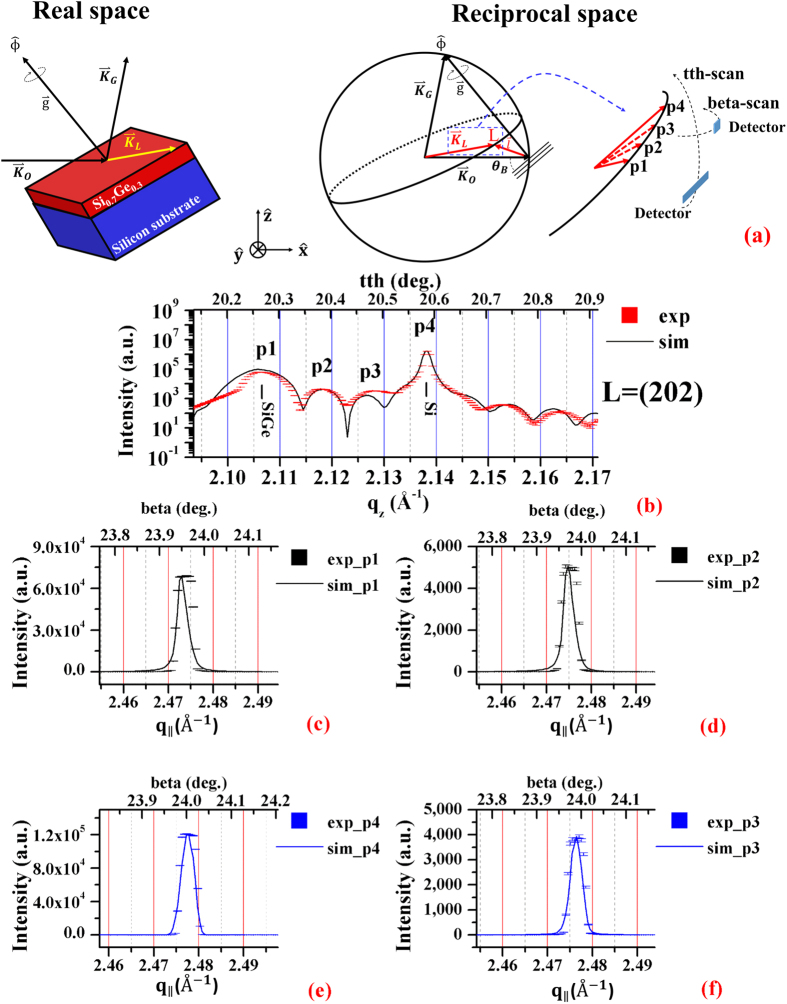
The experiment of Bragg-surface diffraction. (**a**) In the laboratory coordinate, the forward diffracted beam, 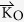, is along the positive x-axis, and is diffracted into 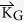 and 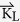 directions. The intensity of the surface diffraction along 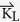 is measured by a tth-scan perpendicular and a beta-scan parallel to the x-y plane of the laboratory coordinate. Peaks, p1 and p4 are the surface diffraction from the thin-film, Si_0.7_Ge_0.3_, and the silicon substrate. Peaks p2 and p3 and the undulating background are the Si_0.7_Ge_0.3_ Kiessig fringes. The slit widths of the vertical tth-scan and horizontal beta-scan are about 10 × 0.1 and 0.1 × 0.1 mm (H × V). (**b**) The measured (red square dot) and simulated (black solid line) tth-scan of the surface diffraction, 202 of the BSD, 004/202, shows the two diffracted peaks, p1 and p4, and the Kiessig fringes including p2 and p3. The top and bottom abscissas are the tth (in deg.) and the corresponding z-component, **q**_z_ (in Å^−1^), of the scattering vector, 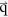 (where the 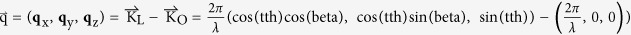, respectively. The four beta-scans were performed at the tth angles: (**c**) p1 (in black), (**d**) p2 (in black), (**e**) p4 (in blue) and (**f**) p3 (in blue), respectively. The top and bottom abscissas are beta (in deg.) and the parallel component, 
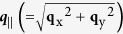
 (in Å^−1^), of the scattering vector, 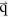, respectively.

**Figure 4:**
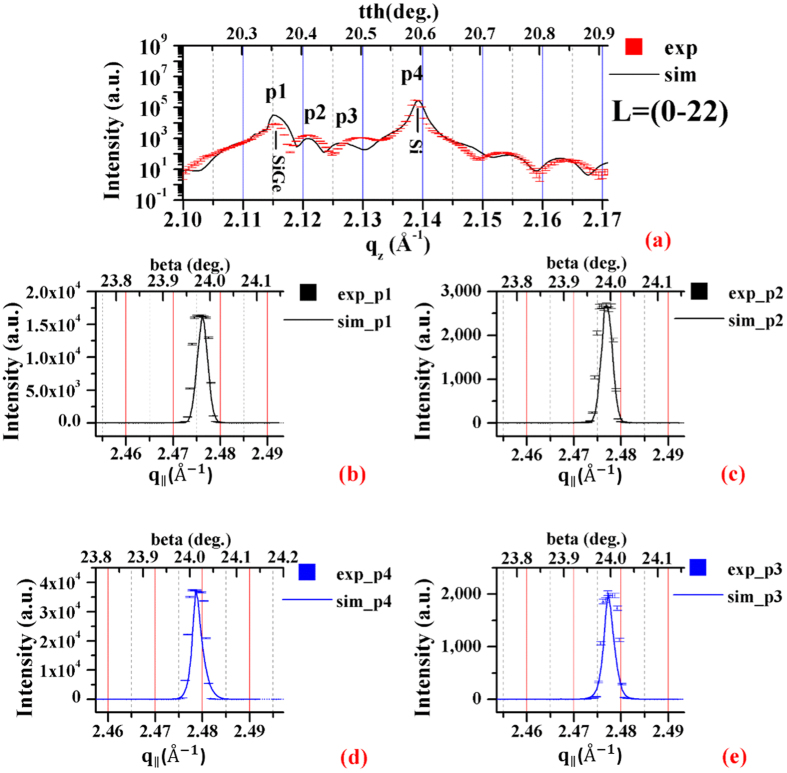
Measured and simulated intensity distributions. (**a**) The simulated (curve in black) and observed (square dot in red) tth-scans (or **q**_z_-scans), are the vertical intensity distribution of the surface diffraction, 0
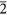
2. The four beta-scans (or 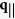-scans) are carried out at the tth angles: (**b**) p1 (in black) (**c**) p2 (in black) (**d**) p4 (in blue) (**e**) p3 (in blue), respectively, with the two abscissas, beta angle (top) and ***q***_||_ (bottom).

**Figure 5:**
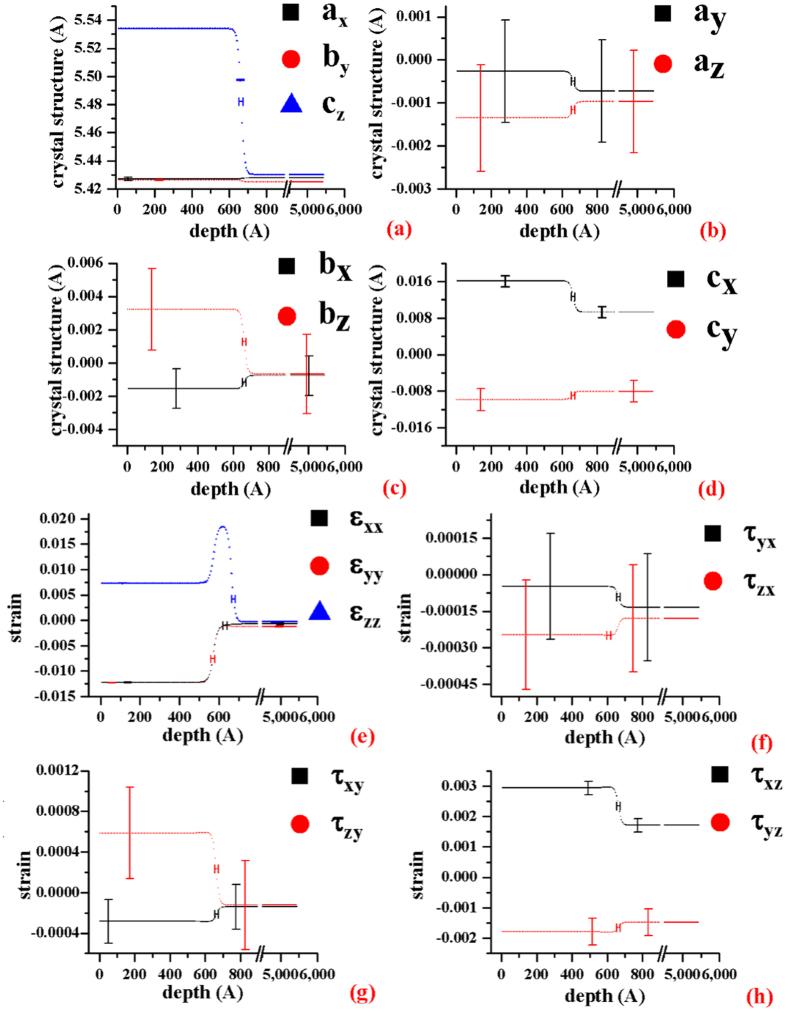
σ-Tensor elements vs. depth. (**a**) The three diagonal elements, a_x_, b_y_ and c_z_ are plotted from the top (depth = 0 Å) to the thin-film, Si_0.7_Ge_0.3_ (until 597 Å), and the interface (597~750 Å), and finally the Si substrate. (**b**) The two elements a_y_ and a_z_ are the y- and z- components of the lattice unit vector, 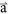. (**c**) The b_x_ and b_z_ are the x- and z-components of the lattice unit vector, 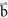. (**d**) The c_x_ and c_y_ are the x- and y- components of the lattice unit vector, 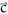. The corresponding strains, calculated according to the predicted lattice constants (See Supplementary Figure 4) from Vegard’s law and the EDS data, Fig. 1c, are: (**e**) the tensile strains, *ε*_xx_, *ε*_yy_ and *ε*_zz_, (**f**) the shear strains, τ_yx_, τ_zx_, (**g**) τ_xy_, τ_zy_, (**h**) τ_xz_, τ_yz_. (See, Supplement IX). The error bars of the ordinates are estimated from the 1^th^ partial derivative of Bragg’s law with respect to tth and beta (see, Supplement VIII). And the error bar of the abscissa is equal to the two times of the average roughness (= 
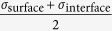
 ~ 1 nm), being about 20 Å. From the Si_0.7_Ge_0.3_ thickness measurements of the EDS (~57 nm, Fig. 2d), HRTEM (~59.9 nm, Fig. 2f), XRR (~58.5 nm, Fig. 2e) and BSD (~59.7 nm, here), the Si_0.7_Ge_0.3_ thickness could be concluded in the range about 57~60 nm.

**Figure 6 f6:**
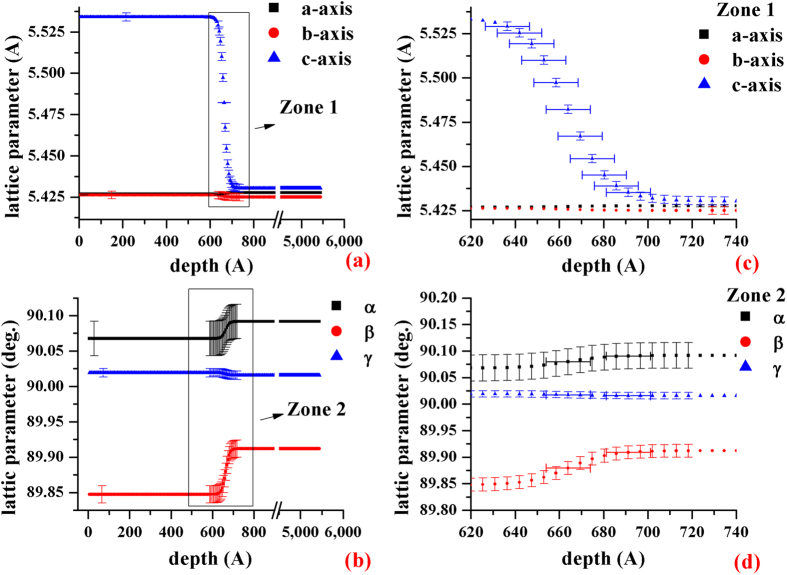
The six lattice parameters versus depth. (**a**) The three lattice parameters, a, b, and c vs depth. (**b**) The three lattice parameters, α, β and γ, vs depth. (**c**) The blow-up of Zone 1 in (**a**), (**d**) The blow-up of Zone 2 of (**b**). The error bars given in figure are estimated from the 1^th^ partial derivative of Bragg’s law with respect to tth and beta (see, Supplement VIII). In the region of the thin-film, Si_0.7_Ge_0.3_ (about 0~597 Å), the six lattice parameters, a, b, c, α, β and γ are about 5.4269 ± 0.00057 Å, 5.426 ± 0.0023 Å, 5.534 ± 0.0024 Å, 90.07 ± 0.024°, 89.85 ± 0.012° and 90.0193 ± 0.0060°, respectively. The lattice parameters at the interface (around 664 Å in depth) are roughly 5.4273 ± 0.0006 Å, 5.425 ± 0.0023 Å, 5.4820 ± 0.0024 Å, 90.080 ± 0.024°, 89.879 ± 0.012° and 90.018 ± 0.006°. That of the silicon substrate (the depths are roughly larger than 745 Å) are approximately 5.42769 ± 0.00057 Å, 5.425 ± 0.0023 Å, 5.430 ± 0.0023 Å, 90.092 ± 0.024°, 89.912 ± 0.012° and 90.016 ± 0.0060°. The c-axis of the thin-film, Si_0.7_Ge_0.3_, is gradually decreased in the interface and reaches the Si value below the interface. While the lattice parameters, a and b, of the Si_0.7_Ge_0.3_ and silicon substrate are nearly the same for all depths.
