# Supplementary material for: Depth profiles of the interfacial strains of Si0.7Ge0.3/Si using three-beam Bragg-surface diffraction
Source: Sci Rep. 2016 May 9;6:25580. doi: 10.1038/srep25580 (PMC4860642; doi:10.1038/srep25580)
Supplement: Supplementary Information [file srep25580-s1.pdf]

Depth profiles of the interfacial strains of $Si_{0.7}Ge_{0.3}$/Si using three-beam Bragg-surface diffraction

Yan-Zong Zheng[1], Yun-Liang Soo[1,2], Shih-Lin Chang[1, 2 *]

[1]*Department of Physics, National Tsing Hua University*

[2]*National Synchrotron Radiation Research Center*

[*]e-mail address: slchang@nsrrc.org.tw; slchang@phys.nthu.edu.tw

I. Estimation of the tensor, $\boldsymbol{\sigma}$

In Real space, the three lattice unit vectors, $\vec{a}$, $\vec{b}$ and $\vec{c}$, of an arbitrary lattice can be described as (a_x, a_y, a_z), (b_x, b_y, b_z) and (c_x, c_y, c_z), which can also be depicted in a tensor, σ (see, Supplementary equation (1)). Similarly, the three reciprocal unit vectors, (a_x^*, a_y^*, a_z^*), (b_x^*, b_y^*, b_z^*) and (c_x^*, c_y^*, c_z^*), can be rewritten as the tensor, $\boldsymbol{\sigma}^*$. The transpose of the inversed tensor, $\boldsymbol{\sigma}^*$ is equal to the tensor, $\boldsymbol{\sigma}$, as

$$\boldsymbol{\sigma} = \begin{bmatrix} a_x & b_x & c_x \\ a_y & b_y & c_y \\ a_z & b_z & c_z \end{bmatrix} = ((\boldsymbol{\sigma}^*)^{-1})^T \qquad (1),$$

where $\boldsymbol{\sigma}^* = \begin{bmatrix} a_x^* & b_x^* & c_x^* \\ a_y^* & b_y^* & c_y^* \\ a_z^* & b_z^* & c_z \end{bmatrix}$.

Because all the diffractions occur in the reciprocal space, the tensor, $\boldsymbol{\sigma}^*$, can be directly measured by means of diffraction techniques. Then the tensor, $\boldsymbol{\sigma}$, can be estimated accordingly (Supplementary equation (1)). As the nine elements of the tensor, σ^*, can be solved by nine equations, and a single reflection, (*h*, *k*, *l*) only provides three equations related to the x-, y- and z-component of its wavevector, say $\vec{K}_L$, therefore given three reflections are sufficient to solve the tensor, $\boldsymbol{\sigma}$. As shown in the main text, the wavevector, $\vec{K}_L$, of the surface diffraction L may be experimentally described by the two peak positions, $2\theta_{peak}$ and β_{peak}, of the tth- and beta-scans. Furthermore, due to the surface refraction and the miscut of Si wafer, the surface wavevector, $\vec{K}_L$, may be corrected by the two values, $\mathbb{N}_1$ and $\mathbb{N}_2$ (for refraction) and the vector, $\vec{R}_m$ (for miscut). Then $\vec{K}_L$ is modified as below.

$$\vec{K}_L = \frac{1}{\lambda}\begin{pmatrix} \mathbb{N}_1 \cos 2\theta_{peak} \cos\beta_{peak} + \mathbb{N}_2 \sin 2\theta_{peak} \\ \cos 2\theta_{peak} \sin\beta_{peak} \\ \mathbb{N}_1 \sin 2\theta_{peak} + \mathbb{N}_2 \cos 2\theta_{peak} \cos\beta_{peak} \end{pmatrix} - \vec{R}_m$$

(2),

where

$\mathbb{N}_1 = \cos\theta_B{}^2 + n \sin\theta_B{}^2$,

$\mathbb{N}_2 = (\bar{n} - 1)\sin\theta_B \cos\theta_B$,

$$\vec{R}_m = \frac{\sin 2\theta_m}{\lambda}\begin{pmatrix}\sin \theta_B \\ 0 \\ \cos \theta_B\end{pmatrix}.$$

The three symbols, θ_B, θ_m and $\bar{n}$ are the Bragg angle, miscut angle with respect to atomic layers and the refractive index. The surface diffraction can be aligned by adjusting the theta angle to the value, θ_B, and by rotating the azimuth, ϕ, around the reciprocal lattice vector, $\vec{g}$ (see, Figure 1b). As the results, the reciprocal lattice vector, $\vec{l}$, of the surface reflection, L, may be rewritten as below

$$\vec{l} = \boldsymbol{T}_y(\theta_B)\boldsymbol{T}_z(\phi)\boldsymbol{\sigma}^*\begin{bmatrix}h \\ k \\ l\end{bmatrix} \quad (3),$$

where

$$\boldsymbol{T}_y(\theta_B) = \begin{bmatrix}\cos\theta_B & 0 & \sin\theta_B \\ 0 & 1 & 0 \\ -\sin\theta_B & 0 & \cos\theta_B\end{bmatrix}, \boldsymbol{T}_z(\phi) = \begin{bmatrix}\cos\phi & -\sin\phi & 0 \\ \sin\phi & \cos\phi & 0 \\ 0 & 0 & 1\end{bmatrix}.$$

The geometry of Figure 1b also indicates the relationship between the three vectors, $\vec{K}_O$, $\vec{l}$ and $\vec{K}_L$, as below:

$$\vec{K}_L = \vec{l} + \vec{K}_O \quad (4),$$

where

$$\vec{K}_O = \begin{bmatrix}x_0 \\ y_0 \\ z_0\end{bmatrix}.$$

The three values, x_0, y_0 and z_0, represent the x-, y- and z-components of the incident wavevector, $\vec{K}_O$. We may replace $\vec{K}_L$ and $\vec{l}$ of Supplementary equation (4) by Supplementary equations (2) and (3), then the three coordinates of Supplementary equation (2) can be written as followings:

$$\begin{cases}\mathbb{C}_0\cos\theta_B\cos\phi - \mathbb{C}_1\cos\theta_B\sin\phi + \mathbb{C}_2\sin\theta_B - x_0 = \frac{\cos 2\theta_{peak}\cos\beta_{peak} - \sin 2\theta_m \sin\theta_B}{\lambda} \\ \mathbb{C}_0\sin\phi + \mathbb{C}_1\cos\phi - y_0 = \frac{\cos 2\theta_{peak}\sin\beta_{peak}}{\lambda} \\ -\mathbb{C}_0\sin\theta_B\cos\phi + \mathbb{C}_1\sin\theta_B\sin\phi + \mathbb{C}_2\cos\theta_B - z_0 = \frac{\sin 2\theta_{peak} - \sin 2\theta_m\cos\theta_B}{\lambda}\end{cases}$$

(5),

where $\mathbb{C}_0 = h\mathrm{a_x}^* + k\mathrm{b_x}^* + l\mathrm{c_x}^*$, $\mathbb{C}_1 = h\mathrm{a_y}^* + k\mathrm{b_y}^* + l\mathrm{c_y}^*$ and $\mathbb{C}_2 = h\mathrm{a_z}^* + k\mathrm{b_z}^* + l\mathrm{c_z}^*$.

Supplementary equation (5) shows that a reflection, (h, k, l) could produce three functions, then the three surface reflections, (202), ($0\bar{2}2$) and ($4\bar{2}2$), can therefore identify the nine components of the tensor, $\boldsymbol{\sigma}$.

II. Introduction to the fundamental equation of X-ray dynamical theory

The dynamical theory of X-ray diffraction begins with Maxwell`s equations,

$$\begin{cases} \vec{\nabla} \times \vec{H} = \left(\frac{\partial}{\partial t}\vec{D} + 4\pi\vec{J}_t\right) \\ \vec{\nabla} \times \vec{E} = -\frac{\partial}{\partial t}\vec{B} \\ \vec{\nabla} \cdot \vec{D} = 4\pi\rho_t \\ \vec{\nabla} \cdot \vec{B} = 0 \end{cases}$$

(6),

where $\vec{E}$, $\vec{D}$, $\vec{H}$ and $\vec{B}$ are the electric field, electric displacement, magnetic field , and magnetic induction, respectively. The two symbols, ρ_t and $\vec{J}_t$, are the given net charge and current density, respectively. In X-ray frequency, the conductivity is about zero, thus the two values, ρ_t and $\vec{J}_t$ are both set to zero. Also the magnetic field, $\vec{H}$, is approximately equal to magnetic induction, $\vec{B}$ due to the magnetic permeability, μ, being about unity. Considering all these conditions mentioned, the Maxwell`s equations can be rewritten as below:

$$\begin{cases} \vec{\nabla} \times \vec{H} = \frac{\partial}{\partial t}\vec{D} \\ \vec{\nabla} \times \vec{E} = -\frac{\partial}{\partial t}\vec{B} \\ \vec{\nabla} \cdot \vec{D} = 0 \\ \vec{\nabla} \cdot \vec{B} = 0 \end{cases}$$

(7).

The Supplementary equation (7) may be deduced as:

$$\left(\nabla^2 + \frac{\partial}{\partial t}\right)\vec{D} = -\left(\vec{\nabla} \times \left(\vec{\nabla} \times \frac{\chi}{(1+\chi)}\vec{D}\right)\right) \approx -\left(\vec{\nabla} \times \left(\vec{\nabla} \times \chi\vec{D}\right)\right)$$

(8).

By considering the periodic nature of the crystal, the assumed solution to Supplementary equation (8) is a Bloch function, namely

$$\vec{D}(\vec{r}, t) = \sum_G \overrightarrow{D_G}\, e^{i(\omega t - 2\pi \vec{k}_G \cdot \vec{r})}$$

(9).

And the electric susceptibility, χ , can be also expressed as a Fourier series as

$$\chi = \sum_m \chi_{h_m} e^{-2\pi i \overrightarrow{h_m} \cdot \vec{r}} \qquad (10).$$

The two symbols, t and $\vec{k}_G$, represent time and the wavevector of the Gth reflection, respectively. The two vectors, $\vec{r}$ and $\vec{r}$, stand for the positions of the electric displacement and susceptibility, respectively. The coefficient, χ_{h_m}, is the electric susceptibility of the ${h_m}^{th}$ reflection.

By substituting Supplementary equations (9) and (10) into (8), the relation among the Fourier components and wavevectors lead to the following so-called fundamental equation of wavefield:

$$(\mathbf{k}_{\boldsymbol{m}} \cdot \mathbf{k}_{\boldsymbol{m}} - K^2)\boldsymbol{E}_{\boldsymbol{m}} = \mathbf{k}_{\boldsymbol{m}}(\mathbf{k}_{\boldsymbol{m}} \cdot \boldsymbol{E}_{\boldsymbol{m}}) + K^2 \sum_{m'}^{N-1} \chi_{m-m'} \boldsymbol{E}_{\boldsymbol{m'}} \qquad (11),$$

where

$\mathbf{k}_{\boldsymbol{m}} = (X_m - x_c)\hat{x} + (Y_m - y_c)\hat{y} + (Z_m - z)\hat{z}$,

and K(=1/λ, λ is the wavelength of the incident beam outside the crystal) is the wave number in vacuum, and m is the mth reflection (m = 0, 1, 2, …and N-1 , N being the N-beam case). Here m = 0 is designated for the incident reflection O. For a three-beam BSD, (O, G, L), m =1 is for the Bragg reflection G and m =2 is for the surface reflection L. $\boldsymbol{E}_{\boldsymbol{m}}$ and $\mathbf{k}_{\boldsymbol{m}}$ are the diffracted E-field and the wavevector inside the crystal of the mth reflection, respectively, The summation $\sum_{m'} \chi_{m-m'}$, is the sum over the Fourier coefficients of the dielectric susceptibility for all the mth reflections. (X_m,Y_m,Z_m) is the reciprocal lattice vector of the mth reflection. The quantities, x_c and y_c, are the x- and y- coordinates of the center of Ewald sphere.

III. Linearization of Eigenvalue problem of the fundamental equation in single Cartesian coordinates [1]

The fundamental equation usually can be solve as an eigenvalue equation, but with quadratic terms of eigenvalues involved. This fact makes numerical calculation difficult. According to Ref. [1] , the use of single Cartesian coordinates can transform the quadratic eigenvalue problem into a linear form, which is easy to solve numerically. Following Ref. [1], a Cartesian coordinates system is chosen where the z-axis, pointing towards the empty space, is normal to the crystal entrance surface. In an N-beam case, in order to describe the diffracted E-fields, $\boldsymbol{E}_{\boldsymbol{m}}$, and wavevectors, $\mathbf{k}_{\boldsymbol{m}}$, the ***m***' reflection with a single Cartesian coordinates system, the N × N diagonal matrices, A, B, and C^S below are utilized to represent the x-, y- and z-component of $\mathbf{k}_{\boldsymbol{m}}$:

$$A \equiv \begin{pmatrix} (X_O - x_c) & 0 & 0 & \cdots & 0 & \\ 0 & (X_G - x_c) & \vdots & \cdots & 0 & \\ \vdots & \vdots & \ddots & \cdots & & 0 \\ \vdots & \vdots & \cdots & (X_m - x_c) & & 0 \\ 0 & 0 & \cdots & \cdots & & \ddots \end{pmatrix}_{N\times N},$$

$$B \equiv \begin{pmatrix} (Y_O - y_c) & 0 & \cdots & 0 \\ 0 & \ddots & \cdots & 0 \\ \vdots & \vdots & (Y_m - y_c) & \vdots \\ 0 & 0 & \cdots & \ddots \end{pmatrix}_{N\times N},$$

$$C^s \equiv \begin{pmatrix} Z_O & 0 & \cdots & 0 \\ 0 & \ddots & \cdots & 0 \\ \vdots & \vdots & Z_m & \vdots \\ 0 & \cdots & 0 & \ddots \end{pmatrix}_{N\times N} - z\begin{pmatrix} 1 & 0 & \cdots & 0 \\ 0 & 1 & \cdots & 0 \\ \vdots & \vdots & \ddots & \vdots \\ 0 & \cdots & 0 & 1 \end{pmatrix} \equiv C - zI \ ,$$

$$\text{where } C = \begin{pmatrix} Z_O & 0 & \cdots & 0 \\ 0 & \ddots & \cdots & 0 \\ \vdots & \vdots & Z_m & \vdots \\ 0 & \cdots & 0 & \ddots \end{pmatrix}_{N\times N} .$$

Also $\boldsymbol{E_m}$ is changed as a 3N × 1 matrix as:

$$\boldsymbol{E_m} = \begin{pmatrix} E_x \\ E_y \\ E_z \end{pmatrix}_{3N\times 1} ,$$

$$E_x = \begin{pmatrix} E_o^x \\ E_G^x \\ \vdots \\ E_m^x \\ \vdots \end{pmatrix}_{N\times 1} , E_y = \begin{pmatrix} E_o^y \\ E_G^y \\ \vdots \\ E_m^y \\ \vdots \end{pmatrix}_{N\times 1} , E_z = \begin{pmatrix} E_o^z \\ E_G^z \\ \vdots \\ E_m^z \\ \vdots \end{pmatrix}_{N\times 1} \qquad (12),$$

where z is the unknown eigenvalue, and (E_m^x, E_m^y, E_m^z) are the x-, y- and z-component of the diffracted E-field of the mth reflection. The matrix form of the fundamental equation then takes the form:

$$[(A^2 + B^2 + (C - zI)^2) - K^2]\begin{pmatrix} E_x \\ E_y \\ E_z \end{pmatrix}$$

$$= \begin{pmatrix} A \\ B \\ (C - zI) \end{pmatrix}\left(AE_x + BE_y + (C - zI)E_z\right) + K^2F\begin{pmatrix} E_x \\ E_y \\ E_z \end{pmatrix} \qquad (13),$$

where $F \equiv \sum_n \chi_{h_m - h_n}$, and $\chi_{h_m - h_n}$ is the electric susceptibility of the $(h_m - h_n)^{\text{th}}$ reflection. Using $G^2 \equiv K^2(I + F)$, with I as a unit matrix, the fundamental equation can be expressed as :

$$\begin{pmatrix} (C - zI)^2 + B^2 - G^2 & -AB & -A(C - zI) \\ -AB & (C - zI)^2 + A^2 - G^2 & -B(C - zI) \\ -A(C - zI) & -B(C - zI) & A^2 + B^2 - G^2 \end{pmatrix}\begin{pmatrix} E_x \\ E_y \\ E_z \end{pmatrix} = 0 \qquad (14).$$

To transform Supplementary equation (14) into a linear eigenvalue problem, two new variables, E_v and E_w, are introduced:

$E_v = (C - zI)E_x - AE_z$

$E_w = (C - zI)E_y - BE_z$

(15).

Finally, the fundamental equation in a linear form is obtained as:

$(\mathrm{Q}-\mathrm{zI})\mathrm{E}_4=0$

(16),

where

$$\mathrm{Q}\equiv\begin{pmatrix} \mathrm{C} & 0 & \mathrm{AG}^{-2}\mathrm{A}-\mathrm{I} & 0 \\ 0 & \mathrm{C} & 0 & \mathrm{BG}^{-2}\mathrm{B}-\mathrm{I} \\ \mathrm{B}^2-\mathrm{G}^2 & -\mathrm{AB} & \mathrm{C} & 0 \\ -\mathrm{AB} & \mathrm{A}^2-\mathrm{G}^2 & 0 & \mathrm{C} \end{pmatrix}_{4N\times4N},$$

$$\mathrm{E}_4=\begin{pmatrix} \mathrm{E_x} \\ \mathrm{E_y} \\ \mathrm{E_v} \\ \mathrm{E_w} \end{pmatrix}_{4N\times4N},\ \mathrm{E_z}=-\mathrm{G}^{-2}(\mathrm{AE_v}+\mathrm{BE_w}),$$

$$\mathrm{E_x}=\begin{pmatrix} \mathrm{E}^{x}_{\mathrm{O1}}\cdots\mathrm{E}^{x}_{\mathrm{Oj}}\cdots\mathrm{E}^{x}_{\mathrm{O4N}} \\ \mathrm{E}^{x}_{\mathrm{G1}}\cdots\mathrm{E}^{x}_{\mathrm{Gj}}\cdots\mathrm{E}^{x}_{\mathrm{G4N}} \\ \vdots \\ \mathrm{E}^{x}_{m1}\cdots\mathrm{E}^{x}_{m\mathrm{j}}\cdots\mathrm{E}^{x}_{m\mathrm{4N}} \\ \vdots \end{pmatrix}_{4N\times4N},\ \mathrm{E_y}=\begin{pmatrix} \mathrm{E}^{y}_{\mathrm{O1}}\cdots\mathrm{E}^{y}_{\mathrm{Oj}}\cdots\mathrm{E}^{y}_{\mathrm{O4N}} \\ \vdots \\ \mathrm{E}^{y}_{m1}\cdots\mathrm{E}^{y}_{m\mathrm{j}}\cdots\mathrm{E}^{y}_{m\mathrm{4N}} \\ \vdots \end{pmatrix}_{4N\times4N},$$

$$\mathrm{E_z}=\begin{pmatrix} \mathrm{E}^{z}_{\mathrm{O1}}\cdots\mathrm{E}^{z}_{\mathrm{Oj}}\cdots\mathrm{E}^{z}_{\mathrm{O4N}} \\ \vdots \\ \mathrm{E}^{z}_{m1}\cdots\mathrm{E}^{z}_{m\mathrm{j}}\cdots\mathrm{E}^{z}_{m\mathrm{4N}} \\ \vdots \end{pmatrix}_{4N\times4N},$$

and $(\mathrm{E}^{x}_{m\mathrm{j}}, \mathrm{E}^{y}_{m\mathrm{j}}, \mathrm{E}^{z}_{m\mathrm{j}})$ is the x-, y- and z-component of the E-field of the jth mode for the mth reflection. The diffracted E-field is then the superposition of the 4N modes with the proportional coefficient, c_j, which is determined though the boundary conditions given in Supplement IV:

$$\vec{E}_m(\vec{r})=\left(\sum_{j=1}^{4N}c_j\vec{E}^{x,y,z}_{mj}e^{-i2\pi\vec{k}_{mj}\cdot\vec{r}}\right)e^{-i2\pi\vec{K}_m\cdot\vec{r}} \qquad (17),$$

where $\vec{k}_{mj}$ is the wavevector inside the crystal of the jth mode for the mth reflection, and $\vec{K}_m$ is the wavevector outside the crystal of the mth reflection, and $\vec{r}$ is the position vector.

IV. Boundary Conditions

For a plane parallel crystal slab (M = 1, where M is the total number of the simulated layers), the coefficient, c_j, can be solved by applying the boundary conditions, i.e., the continuity of the tangential components of the electric field, $\vec{E}$,

and magnetic field, $\vec{H}$, and of the normal components of the electric displacement, $\vec{D}$, and magnetic induction, $\vec{B}$, at the top (l = 1) and bottom (l = 2) boundaries. These lead to the following equations:

$$\sum_{j=1}^{4N} c_j E_{mj}^{(n)x,y} \phi_{mj(l)}^{(n)} = \hat{E}_m^{(n)x,y} + \delta_{m(l)}^{(n)} \tilde{E}^{x,y} \tag{18}$$

$$\sum_{j=1}^{4N} c_j \left(z_{mj}^{(n)} E_{mj}^{(n)y} - y_m^{(n)} E_{mj}^{(n)z} \right) \phi_{mj(l)}^{(n)}$$

$$= (-1)^l K_{m(l)}^{(n)z} \hat{E}_{m(l)}^{(n)y} + y_m^{(n)} \hat{E}_{m(l)}^{(n)z} + \delta_{m(l)}^{(n)} \left(K_{m(l)}^{z(1)} \tilde{E}^y - y_m^{(1)} \tilde{E}^z \right) \tag{19}$$

$$\sum_{j=1}^{4N} c_j \left(E_{mj}^{(n)z} + \sum_{n'=0}^{N-1} \chi_{m-n'}^{(n)} E_{n'j}^{(n)z} \right) \phi_{mj(l)}^{(n)} = \hat{E}_{m(l)}^{(n)z} + \delta_{m(l)}^{(n)} \tilde{E}^z \tag{20}$$

$$\sum_{j=1}^{4N} c_j \left(x_m^{(n)} E_{mj}^{(n)z} - z_{mj}^{(n)} E_{mj}^{(n)x} \right) \phi_{mj(l)}^{(n)}$$

$$= x_m^{(n)} \hat{E}_{m(l)}^{(n)z} - (-1)^l K_{m(l)}^{(n)z} \hat{E}_{m(l)}^{(n)x} + \delta_{m(l)}^{(n)} \left(x_m^{(1)} \tilde{E}^z - K_{m(l)}^{(1)z} \tilde{E}^x \right) \tag{21},$$

where

$x_m^{(n)} = X_m^{(n)} - x_c$, $y_m^{(n)} = Y_m^{(n)} - y_c$,

$\phi_{mj(l)}^{(n)} = e^{i2\pi z_{mj}^{(n)} t_{(l)}^{(n)}}$, and $z_{mj}^{(n)} = Z_m^{(n)} - z_j^{(n)}$.

In Supplementary equations (18)-(21), ($X_m^{(n)}$, $Y_m^{(n)}$, $Z_m^{(n)}$) is the reciprocal lattice vector of the mth reflection at the nth atomic layer, and the vector, ($\tilde{E}^x$, $\tilde{E}^y$, $\tilde{E}^z$), is the incident E-field, the term, ($\hat{E}_{m(l)}^{(n)x}$, $\hat{E}_{m(l)}^{(n)y}$, $\hat{E}_{m(l)}^{(n)z}$), is the mth reflection`s diffractive E-field at the top (l = 1) or bottom (l = 2) of the nth atomic layer, and the z-component of the wavevector, $K_{m(l)}^{(n)z}$, outside the crystal is given below:

$$K_{m(l)}^{(n)z} = \begin{cases} -\left(\sqrt{\left(\frac{1}{\lambda}\right)^2 - \left[\left(x_m^{(n)}\right)^2 + \left(y_m^{(n)}\right)^2 \right] \times (\cos(\theta_m))^2} + \frac{1}{\lambda}\sin(\theta_m) \right) & \text{when } l = 1 \\ -\sqrt{\left(\frac{1}{\lambda}\right)^2 - \left(x_m^{(n)}\right)^2 - \left(y_m^{(n)}\right)^2} & \text{when } l = 2\text{'} \end{cases}$$

$$\delta_{m(l)}^{(n)} = \begin{cases} 1 & m = 0, l = 1 \text{ and } n = 1 \\ 0 & \text{otherwise} \end{cases},$$

$$t_{(l)}^{(n)} = \begin{cases} \begin{cases} 0 & l = 1 \\ t & l = 2 \end{cases} & \text{for plane parallel crystal slab} (\mathrm{M} = 1) \\ \begin{cases} \mathrm{t}_{(1)}^{(n)} & l = 1 \\ \mathrm{t}_{(2)}^{(n)} & l = 2 \end{cases} & \text{for multi layer } (\mathrm{M} > 1) \end{cases},$$

$$\mathrm{t}^{(n)} = \mathrm{t}_{(2)}^{(n)} - \mathrm{t}_{(1)}^{(n)},$$

where the symbol, *t*, is the thickness of the parallel crystal slab. For a multi atomic layers (Supplement V), $\mathrm{t}_{(1)}^{(n)}$ and $\mathrm{t}_{(2)}^{(n)}$ are the top and bottom of the nth atomic layer, and $\mathrm{t}^{(n)}$ is the thickness of the nth atomic layer.

V. Multi-layer dynamical theory of X-ray diffraction form crystalline [2]

Because the sample system, $Si_{0.7}Ge_{0.3}/Si$, is composed of many atomic layers with continually changing lattice parameters, the ratios among the E-fields, $\vec{E}_m^{(n)}$, and the wavevector, $\vec{k}_m^{(n)}$, of each layer, say the nth layer for the mth reflection can be firstly calculated by the eigenvalue equation, Supplementary equation (16). For a multilayer system, the coefficients, $c_j^{(n)}$, can be determined simultaneously from the boundary conditions at the top and bottom surface, and the intermediate n^{th}-to-$(n+1)^{\mathrm{th}}$ boundary of the sample by using respectively the Supplementary equations (18~21) and Supplementary equations (22~25) given below, where $(x_m^{(n)}, y_m^{(n)}, z_{mj}^{(n)})$ is the diffracted wavevector inside the crystal due to the variation of lattice parameters from layer to layer:

$$\sum_{j=1}^{4N} c_j^{(n)} E_{mj}^{(n)x,y} \phi_{mj(2)}^{(n)} = \sum_{j=1}^{4N} c_j^{(n+1)} E_{mj}^{(n+1)x,y} \phi_{mj(1)}^{(n+1)} \tag{22}$$

$$\sum_{j=1}^{4\mathrm{N}} c_j^{(n)} \left(z_{mj}^{(n)} E_{mj}^{(n)y} - y_m^{(n)} E_{mj}^{(n)z} \right) \phi_{mj(2)}^{(n)}$$

$$= \sum_{j=1}^{4N} c_j^{(n+1)} \left(z_{mj}^{(n+1)} E_{mj}^{(n)y} - y_m^{(n+1)} E_{mj}^{(n+1)z} \right) \phi_{mj(1)}^{(n+1)} \tag{23}$$

$$\sum_{j=1}^{4N} c_j^{(n)} \left(E_{mj}^{(n)z} + \sum_{n'=0}^{N-1} \chi_{m-n'}^{(n)} E_{n'j}^{(n)z} \right) \phi_{mj(2)}^{(n)}$$

$$= \sum_{j=1}^{4N} c_j^{(n+1)} \left(E_{mj}^{(n+1)z} + \sum_{n'=0}^{N-1} \chi_{m-n'}^{(n+1)} E_{n'j}^{(n+1)z} \right) \phi_{mj(1)}^{(n+1)} \quad (24)$$

$$\sum_{j=1}^{4N} c_j^{(n)} \left(x_m^{(n)} E_{mj}^{(n)z} - z_{mj}^{(n)} E_{mj}^{(n)x} \right) \phi_{mj(l)}^{(n)}$$

$$= \sum_{j=1}^{4N} c_j^{(n+1)} \left(x_m^{(n)} E_{mj}^{(n)z} - z_{mj}^{(n)} E_{mj}^{(n)x} \right) \phi_{mj(l)}^{(n)} \quad (25),$$

In the Supplementary equations (22~25), ($E_{mj}^{(n)x}, E_{mj}^{(n)y}, E_{mj}^{(n)z}$) and ($x_m^{(n)}, y_m^{(n)}, z_{mj}^{(n)}$) stand for the electric field and its wavevector of the jth mode for the *m* reflection at the nth layer inside the crystal. To solve the coefficients, $c_j^{(n)}$, for the top and bottom surface of the multilayer sample, the calculations of Supplementary equations (18~22) lead to the following matrix equations:

$$\begin{cases} \bar{A}_{(1)}^{(1)} \Psi_{(1)}^{(1)} C^{(1)} = \bar{E}_0 \\ \bar{B}_{(2)}^{(\mathrm{M})} \Psi_{(2)}^{(\mathrm{M})} C^{(\mathrm{M})} = \bar{0} \end{cases}$$

(26),

where

$$C^{(n)} = \left(c_1^{(n)} c_2^{(n)} \cdots c_j^{(n)} \cdots c_{4N}^{(n)} \right)^T ,$$

$$\bar{A}_{(1)}^{(1)} = \begin{pmatrix} \bar{a}_{(1)}^{(1)x} \phi_{m(1)}^{(1)} \\ \bar{a}_{(1)}^{(1)y} \phi_{m(1)}^{(1)} \end{pmatrix}_{2N\times 4N}, \bar{B}_{(2)}^{(M)} = \begin{pmatrix} \bar{b}_{(2)}^{(M)x} \phi_{m(2)}^{(\mathrm{M})} \\ \bar{b}_{(2)}^{(M)y} \phi_{m(2)}^{(\mathrm{M})} \end{pmatrix}_{2N\times 4N} \text{ and } \bar{E}_0 = \begin{pmatrix} e^x \\ 0 \\ \vdots \\ e^y \\ 0 \\ \vdots \\ 0 \end{pmatrix}_{2N\times 1},$$

$$\bar{a}_{(1)}^{(1)\binom{x}{y}} = \left(z_{mj}^{(1)} + K_{m(1)}^{(1)z} \right) \begin{pmatrix} E_{mj}^{(1)x} \\ E_{mj}^{(1)y} \end{pmatrix} + \begin{pmatrix} x_m^{(1)} \\ y_m^{(1)} \end{pmatrix} \sum_{m'=0}^{N-1} \chi_{m-m'}^{(1)} E_{m'j}^{(1)z} ,$$

$$\bar{b}_{(2)}^{(M)\binom{x}{y}} = \left(z_{mj}^{(M)} - K_{m(2)}^{(M)z} \right) \begin{pmatrix} E_{mj}^{(M)x} \\ E_{mj}^{(M)y} \end{pmatrix} + \begin{pmatrix} x_m^{(M)} \\ y_m^{(M)} \end{pmatrix} \sum_{m'=0}^{N-1} \chi_{m-m'}^{(M)} E_{m'j}^{(M)z} .$$

In Supplementary equation (26), we choose the total number of the simulated atomic layers, M, equal to 1000. The symbol, c_j, of Supplementary equations (18~22) are changed as $c_j^{(n)}$ for dealing with the multi-layer calculation. Similarly, the

calculations of Supplementary equations (22~25) for the intermediate boundary give the matrix equation:

$$\bar{D}_{(l)}^{(n)}\Psi_{(n)}^{(n)}C^{(n)} = \bar{F}_{(l)}^{(n+1)}\Psi_{(n+1)}^{(n+1)}C^{(n+1)} \quad (27),$$

where $\bar{D}_{(l)}^{(n)} = \begin{pmatrix} \bar{d}_{(l)}^{(n)x}\phi_{m(l)}^{(n)} \\ \bar{d}_{(l)}^{(n)y}\phi_{m(l)}^{(n)} \end{pmatrix}$ and $\bar{F}_{(l)}^{(n+1)} = \begin{pmatrix} \bar{f}_{(l)}^{(n+1)x}\phi_{m(l)}^{(n+1)} \\ \bar{f}_{(l)}^{(n+1)y}\phi_{m(l)}^{(n+1)} \end{pmatrix}$,

$$\bar{d}_{(l)}^{(n)\binom{x}{y}} = \left(z_{mj}^{(n)} - (-1)^l K_{m(l)}^{(n+1)z} -\right)\begin{pmatrix} E_{mj}^{(n)x} \\ E_{mj}^{(n)y} \end{pmatrix}$$

$$-\begin{pmatrix} x_m^{(n)} - x_m^{(n+1)} \\ y_m^{(n)} - y_m^{(n+1)} \end{pmatrix} E_{mj}^{(n)z} + \begin{pmatrix} x_m^{(n+1)} \\ y_m^{(n+1)} \end{pmatrix} \sum_{m'=0}^{N-1} \chi_{m-m'}^{(n)} E_{m'j}^{(n)z} \ ,$$

$$\bar{f}_{(l)}^{(n)\binom{x}{y}} = \left(z_{mj}^{(n)} - (-1)^l K_{m(l)}^{(n)z}\right)\begin{pmatrix} E_{mj}^{(n)x} \\ E_{mj}^{(n)y} \end{pmatrix} + \begin{pmatrix} x_m^{(n)} \\ y_m^{(n)} \end{pmatrix} \sum_{m'=0}^{N-1} \chi_{m-m'}^{(n)} E_{m'j}^{(n)z} .$$

To avoid the divergence of $\phi_{mj(l)}^{(n)}$ at deeper depth, $c_j^{(n)}$ is written with a different unknown proportional coefficient, a phase factor:

$$C^{(n)} = \mathrm{S}^{(n)}\hat{C}^{(n)} \quad (28),$$

where

$$\hat{C}^{(n)} = \left(\hat{c}_1^{(n)}\ \hat{c}_2^{(n)} \cdots \hat{c}_{4\mathrm{N}}^{(n)}\right)^T ,$$

, and a conditional coefficient S:

$$[\mathrm{S}^{(n)}]_{jj} = \begin{cases} \left.\begin{matrix} 1 & imag\left(z_j^{(n)}\right) < 0 \\ e^{i2\pi z_j^{(n)} t^{(n)}} & imag\left(z_j^{(n)}\right) > 0 \end{matrix}\right\} l = 1 \\ \left.\begin{matrix} e^{-i2\pi z_j^{(n)} t^{(n)}} & imag\left(z_j^{(n)}\right) < 0 \\ 1 & imag\left(z_j^{(n)}\right) > 0 \end{matrix}\right\} l = 2 \end{cases} .$$

By combining Supplementary equations (26~28) , the following equations are obtained:

$$\begin{cases} D_{mid}^{(1)}\hat{C}^{(1)} = E_0 + F_{(1)}^{(2)} \\ D_{mid}^{(n)}\hat{C}^{(n)} = D_{(2)}^{(n)}\hat{C}^{(n)} + F_{(1)}^{(n+1)}\hat{C}^{(n+1)} \\ D_{mid}^{(\mathrm{M})}\hat{C}^{(\mathrm{M})} = D_{(2)}^{(\mathrm{M}-1)}\hat{C}^{(\mathrm{M}-1)} \end{cases}$$

(29),

$$\text{where } D_{mid}^{(n)} = \begin{cases} A_{(1)}^{(1)} + D_{(2)}^{(1)} & \text{the } 1^{th} \text{ layer} \\ D_{(1)}^{(n)} + F_{(2)}^{(n)} & \text{the } n^{th} \text{ layer} \\ F_{(1)}^{(\mathrm{M})} + B_{(2)}^{(\mathrm{M})} & \text{the } \mathrm{M}^{th} \text{ layer} \end{cases}$$

$$E_0 = \begin{pmatrix} \bar{E}_0 \\ \bar{0} \end{pmatrix}, A_{(l)}^{(n)} = \begin{pmatrix} \bar{A}_{(l)}^{(n)} S_{(l)}^{(n)} \\ \bar{0} \end{pmatrix} \text{ and } B_{(l)}^{(n)} = \begin{pmatrix} \bar{0} \\ \bar{B}_{(l)}^{(n)} S_{(l)}^{(n)} \end{pmatrix}$$

$$D_{(l)}^{(n)} = \begin{pmatrix} \bar{0} \\ \bar{D}_{(l)}^{(n)} S_{(l)}^{(n)} \end{pmatrix} \text{ and } F_{(l)}^{(n)} = \begin{pmatrix} \bar{F}_{(l)}^{(n)} S_{(l)}^{(n)} \\ \bar{0} \end{pmatrix}.$$

The symbol, $\bar{0}$, is a 2N×4N null matrix.

The Supplementary equation (29) can be solved as a linear matrix equation as:

$$\mathbb{Q} \times \mathbb{C} = \boldsymbol{E} \qquad (30),$$

where

$$\mathbb{Q} = \begin{pmatrix} \boldsymbol{D}_{mid}^{(1)} & -\boldsymbol{F}_{(1)}^{(2)} & \boldsymbol{O} & \boldsymbol{O} & \boldsymbol{O} & \boldsymbol{O} & \cdots & \boldsymbol{O} \\ -\boldsymbol{D}_{(2)}^{(1)} & \boldsymbol{D}_{mid}^{(2)} & -\boldsymbol{F}_{(1)}^{(3)} & \boldsymbol{O} & \boldsymbol{O} & \boldsymbol{O} & \cdots & \boldsymbol{O} \\ \boldsymbol{O} & \boldsymbol{O} & \ddots & \boldsymbol{O} & \boldsymbol{O} & \vdots & \boldsymbol{O} & \boldsymbol{O} \\ \boldsymbol{O} & \boldsymbol{O} & -\boldsymbol{D}_{(2)}^{(n-2)} & \boldsymbol{D}_{mid}^{(\mathrm{n}-1)} & -\boldsymbol{F}_{(1)}^{(n)} & \boldsymbol{O} & \vdots & \vdots \\ \boldsymbol{O} & \boldsymbol{O} & \boldsymbol{O} & -\boldsymbol{D}_{(2)}^{(n-1)} & \boldsymbol{D}_{mid}^{(\mathrm{n})} & -\boldsymbol{F}_{(1)}^{(n+1)} & \boldsymbol{O} & \boldsymbol{O} \\ \vdots & \vdots & \vdots & \boldsymbol{O} & \vdots & \ddots & \ddots & \boldsymbol{O} \\ \boldsymbol{O} & \boldsymbol{O} & \boldsymbol{O} & \boldsymbol{O} & \boldsymbol{O} & -\boldsymbol{D}_{(2)}^{(\mathrm{M}-2)} & \boldsymbol{D}_{mid}^{(\mathrm{M}-1)} & -\boldsymbol{F}_{(1)}^{(\mathrm{M})} \\ \boldsymbol{O} & \boldsymbol{O} & \boldsymbol{O} & \boldsymbol{O} & \cdots & \boldsymbol{O} & \boldsymbol{D}_{(2)}^{(\mathrm{M}-1)} & \boldsymbol{D}_{mid}^{(\mathrm{M})} \end{pmatrix},$$

$$\mathbb{C} = (\hat{C}^{(1)}, \hat{C}^{(2)}, \cdots, \hat{C}^{(n-1)}, \hat{C}^{(n)}, \hat{C}^{(n+1)}, \cdots, \hat{C}^{(M-2)}, \hat{C}^{(M-1)}, \hat{C}^{(M)})^T$$

, and $\boldsymbol{E} = (E_0, O, \cdots, O)^T$.

In Supplementary equation (30), the dimensions of the matrices are (4N×M)×(4N×M) for $\mathbb{Q}$, (4N×M)×4N for both $\mathbb{C}$ and $\boldsymbol{E}$, and 4N×4N for the null matrix O. The unknown coefficients, $\mathbb{C}$ can be solved as:

$$\mathbb{C} = \mathbb{Q}^{-\mathbf{1}} \boldsymbol{E} \qquad (31).$$

Because the size of the matrix, $\mathbb{Q}$, is so huge that its inverse matrix cannot be estimated in the general software due to the problem, "out of memory". However, using the method given in Ref. [3], the inversed matrix of $\mathbb{Q}$ can be solved. Then, the elements, $\hat{c}^{(n)}$, of the matrix $\mathbb{C}$ are determined:

$$\hat{C}^{(n)} = \prod_j^n Q^{(\mathrm{j})} \left[D_{mid}^{(1)} - \Gamma^{(2)} \right]^{-1} \overline{E_0} \qquad (32),$$

where

$$Q^{(M-\mathrm{j})} = \left[D_{mid}^{(M-\mathrm{j})} - \Gamma^{(M-\mathrm{j})} \right]^{-1} D_{(2)}^{(M-\mathrm{j}-1)},$$

$$\Gamma^{(M-\mathrm{j})} = F_{(1)}^{(M-\mathrm{j}+1)} \left[D_{mid}^{(M-\mathrm{j}+1)} - \Gamma^{(M-\mathrm{j}+1)} \right]^{-1} D_{(2)}^{(M-\mathrm{j})} \text{ when } 1 \le \mathrm{j} \le (\mathrm{M}-1).$$

Finally, the nth layer`s diffracted E-field of the mth reflection is described as:

$$\vec{E}_{m(l)}^{(n)} = \left(\sum_{j=1}^{4N} \phi_{m(l)}^{(n)} S_{(l)}^{(n)} \hat{c}_j^{(n)} E_{mj}^{(n)x,y,z}\right) e^{-i2\pi \vec{K}_{m(l)}^{(n)} \cdot \vec{r}_{(l)}^{(n)}} \qquad (33),$$

where

$\phi_{ml}^{(n)} = e^{i2\pi Z_m^{(n)} t_{(l)}^{(n)}}$, $\vec{K}_{m(l)}^{(n)} = (x_m^{(n)}, y_m^{(n)}, (-1)^l K_{m(l)}^{(n)z})$, and $\vec{r}_{(l)}^{(n)} = (0,0,-t_{(l)}^{(n)})$.

VI. Construction of fitting function, $\mathcal{F}_m^{(n)}\left(\vec{R}\ (\text{tth}, \text{beta})\right)$

A fitting function, $\mathcal{F}_m^{(n)}\left(\vec{R}\ (\text{tth}, \text{beta})\right)$, is to simulate the intensity distributions of the surface diffraction, *m*, at the nth layer, which is monitored by a detector. Because of the shallow diffraction angle of the surface reflection, *m*, with respect to the crystal surface, part of the diffracted beam is absorbed by the crystal. The acceptance of the detector depends therefore on the diffraction geometry, crystal orientation, crystal depth, beam divergence, and the miscut angle between the surface and atomic layers. For better handling the simulated intensity, we use the measured tth- and beta-scans to construct a fitting function of the nth layer. Consequently, the surface wavevector (l = 1), $\vec{K}_{m(l)}^{(n)}$ $(=(x_m^{(n)}, y_m^{(n)}, -K_{m(1)}^{(n)z}))$, should be firstly transferred to our laboratory tth-beta coordinate system by rotating the crystal a Bragg angle, θ_B, around the y-axis, so that the incident beam is along the x-axis. The surface wavevector, $-\vec{K}_{m(1)}^{(n)}$, denoted as ($K^{(n)x}$, $K^{(n)y}$, $K^{(n)z}$), in the laboratory coordinate system, then takes the form as:

$$\begin{pmatrix} K^{(n)x} \\ K^{(n)y} \\ K^{(n)z} \end{pmatrix} = \begin{pmatrix} \cos\theta_B & 0 & \sin\theta_B \\ 0 & 1 & 0 \\ -\sin\theta_B & 0 & \cos\theta_B \end{pmatrix} \begin{pmatrix} x_m^{(n)} \\ y_m^{(n)} \\ -K_{m(1)}^{(n)z} \end{pmatrix},$$

which describes the surface wavevector in tth-beta coordinate system by the two angles, $2\theta_c^{(n)}$ and $\beta_c^{(n)}$, defined as:

$2\theta_c^{(n)} = \sin^{-1}(\lambda K^{(n)z})$,

$$\beta_c^{(n)} = \begin{cases} \cos^{-1}\left(\frac{K^{(n)x}}{\sqrt{\left(K^{(n)x}\right)^2+\left(K^{(n)y}\right)^2}}\right) & \text{when } K^{(n)y} \geq 0 \\ 180° + \cos^{-1}\left(\frac{K^{(n)x}}{\sqrt{\left(K^{(n)x}\right)^2+\left(K^{(n)y}\right)^2}}\right) & \text{when } K^{(n)y} < 0 \end{cases}.$$

Because the sample is composed of the thin-film, $Si_{0.7}Ge_{0.3}$, interface and silicon substrate, the fitting function, $\mathcal{F}_m^{(n)}\left(\vec{R}\,(\text{tth},\text{beta})\right)$ can be written as

$$\mathcal{F}_m^{(n)}\left(\vec{R}\,(\text{tth},\text{beta})\right)$$

$$= F_m^{(n)\text{SiGe}}\left(\vec{R}\left(\text{tth} - 2\theta_c^{(n)}, \text{beta} - \beta_c^{(n)}\right)\right) \times CS^{(n)\text{SiGe}}$$

$$+F_m^{(n)\text{Si}}\left(\vec{R}(\text{tth} - 2\theta_c^{(n)}, \text{beta} - \beta_c^{(n)})\right) \times CS^{(n)\text{Si}} \quad (34),$$

where $F_m^{(n)\text{SiGe}}$ and $F_m^{(n)\text{Si}}$ are both linear combinations of the Gaussian, Lorentz, Laplace, and asymmetric functions, which fit the experimental tth- and beta-scans with the peak positions at $2\theta_c^{(n)}$ and $\beta_c^{(n)}$, respectively. $CS^{(n)\text{SiGe}}$ and $CS^{(n)\text{Si}}$ are the composition ratios of SiGe and Si at the nth layer, which act as weighting factors (See, Supplementary Figure 3). The fitting functions $F_m^{(n)\text{SiGe}}$ and $F_m^{(n)\text{Si}}$ for the surface reflections, (202), $(0\bar{2}2)$, $(4\bar{2}2)$, and $(2\bar{4}2)$, are shown in Supplementary Figure 1 and Figure 2, respectively. For unifying the two coordinates, tth-beta and eigenvalue-eigenvector, of the equation (3), the position vector $\vec{R}\,(\text{tth},\text{beta})$ of the detector from the sample is used an given as:

$$\vec{R}(\text{tth},\text{beta}) = R\begin{pmatrix} \cos\theta_B & 0 & -\sin\theta_B \\ 0 & 1 & 0 \\ \sin\theta_B & 0 & \cos\theta_B \end{pmatrix}\begin{pmatrix} \cos(\text{tth})\cos(\text{beta}) \\ \cos(\text{tth})\sin(\text{beta}) \\ \sin(\text{tth}) \end{pmatrix} \quad (35),$$

where R is the distance between the detector and sample.

VII. Results of fitting function, $\mathcal{F}_m^{(n)}\left(\vec{\boldsymbol{R}}\,(\mathbf{tth},\mathbf{beta})\right)$

Here we show the fitting functions when $2\theta_c^{(n)}$ and $\beta_c^{(n)}$ are both equal to zero in Supplementary Figure 1 and Figure 2. As can be seen, the full widths at half maxima (FWHM) of Supplementary Figure 1 are larger than that of Supplementary Figure 2 due to the fact that the crystal quality of Si substrate is better than that of SiGe. Also, the profile of the vertical (tth) direction in Supplementary Figure 1 is asymmetric with respect to the central position. The reason is that the diffracted wavevector of the thin-film, $Si_{0.7}Ge_{0.3,}$ is almost parallel to the surface of the sample so that the lower half of the profile is absorbed drastically by the crystal, however, the diffracted wavevector of the silicon substrate is above the sample surface, and thus the profile is approximately symmetric about the central position.

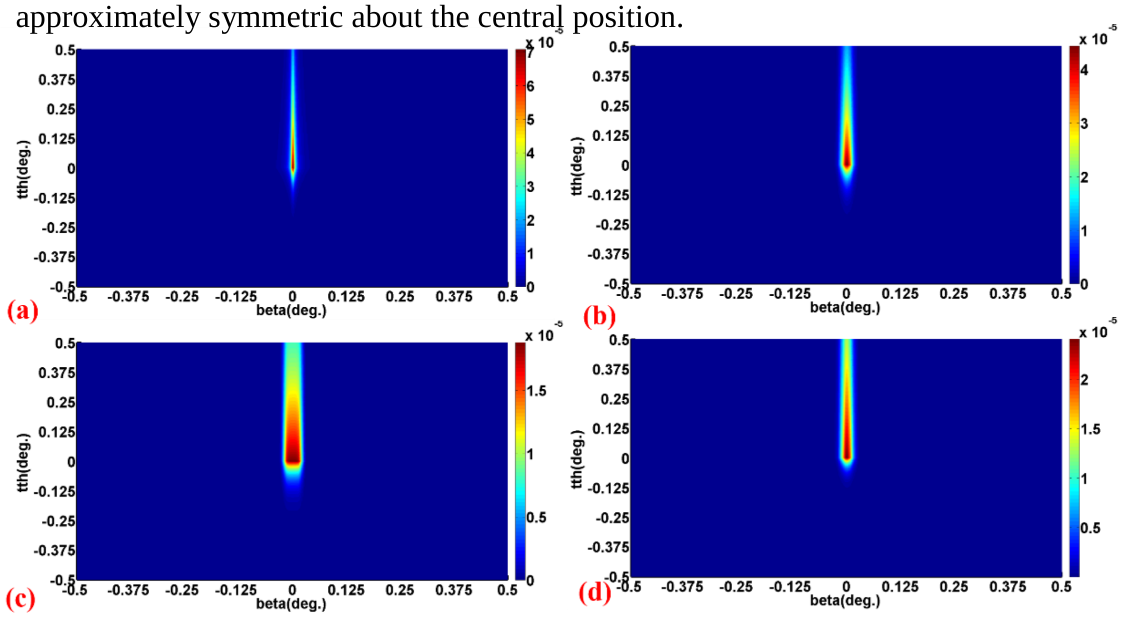

Figure 1 | Fitting Function, $F_m^{(n)\mathrm{SiGe}}$ **(a)** (202) **(b)** $(0\bar{2}2)$ **(c)** $(4\bar{2}2)$ **(d)** $(2\bar{4}2)$

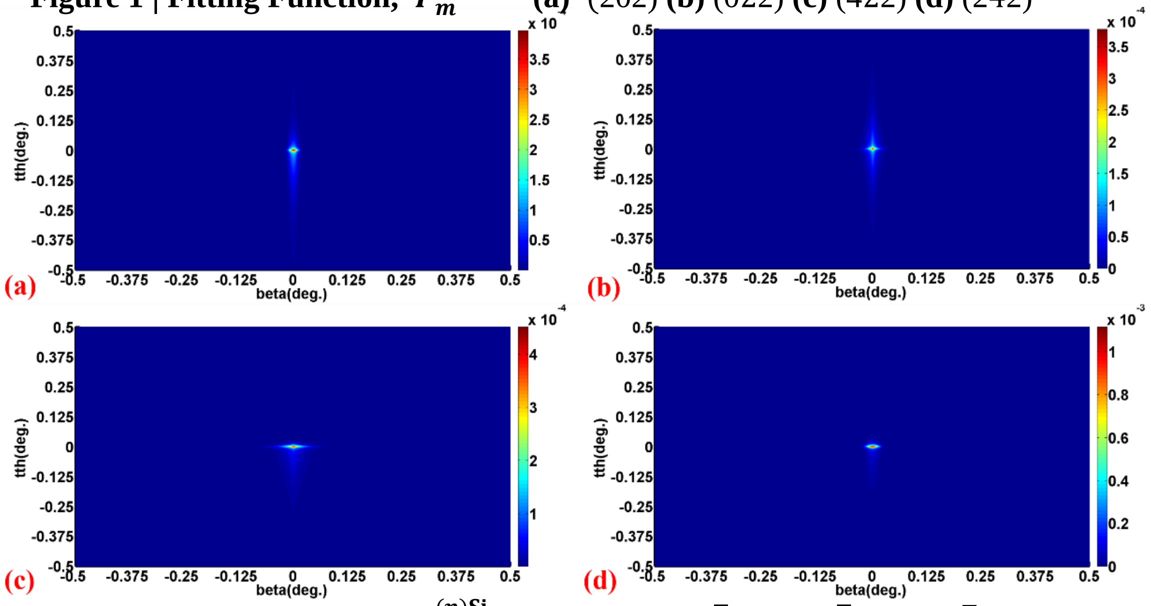

Figure 2 | Fitting Function, $F_m^{(n)\mathrm{Si}}$ **(a)** (202) **(b)** $(0\bar{2}2)$ **(c)** $(4\bar{2}2)$ **(d)** $(2\bar{4}2)$

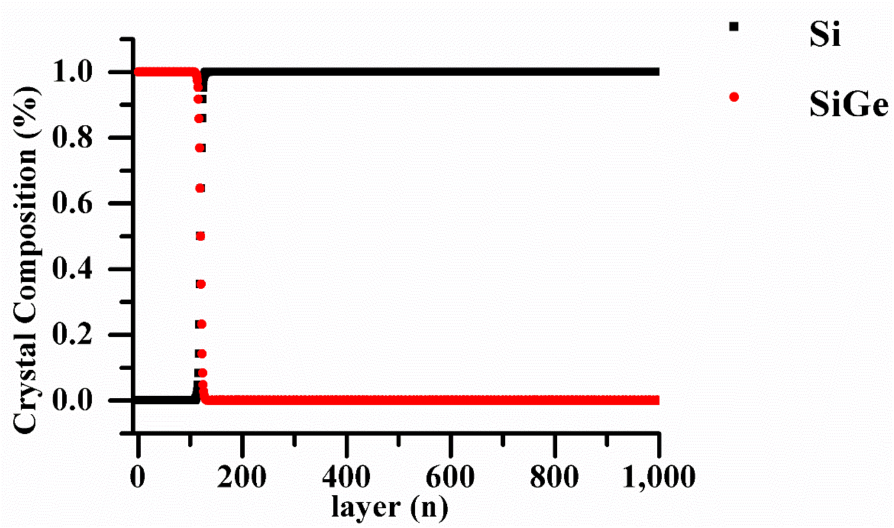

Figure 3 | Crystal compositions Vs. depth The black square dot is the function, $CS^{(n)\mathrm{SiGe}}$ and the function, $CS^{(n)\mathrm{Si}}$ is the red circle dot.

VIII. Evaluation the uncertainties of the σ tensor and lattice parameters

In the tth- and beta-scans, the slit width of the detector may cause uncertainties in measuring the σ tensor and lattice parameters. The Bragg`s law can be written in relation to the lattice parameters and σ tensor of the nth layer as:

$$\frac{2\sin{\Theta_B}^{(n)}}{\lambda} = d^{*(n)} = \left|\Omega^{(n)}\begin{bmatrix} h \\ k \\ l \end{bmatrix}\right|$$

(36),

where

$$\Omega^{(n)} = \begin{cases} \begin{bmatrix} {a_x}^* & {b_x}^* & {c_x}^* \\ {a_y}^* & {b_y}^* & {c_y}^* \\ {a_z}^* & {b_z}^* & c_z \end{bmatrix}^{(n)} & \text{in terms of } \sigma \text{ tensor} \\ \begin{bmatrix} a^* & b^*\cos\gamma^* & c^*\cos\beta^* \\ 0 & b^*\sin\gamma^* & -c^*\sin\beta^*\cos\alpha \\ 0 & 0 & -c^*\sin\beta^*\sin\alpha \end{bmatrix}^{(n)} & \text{in terms of lattice parameters} \end{cases},$$

and $d^{*(n)}$ is the reciprocal lattice spacing of the nth layer, and the six symbols, and a^*, b^*, c^*, α^*, β^* and γ^*, are the six parameters in reciprocal space. The Bragg angle can be expressed as ${\Theta_B}^{(n)} = \cos^{-1}\frac{\left(\cos\left(2\theta_c^{(n)}\right)\cos\left(\beta_c^{(n)}\right)\right)}{2}$, the 1th order derivative of Bragg`s law with respect to tth and beta leads to the following variation equation:

$$-\frac{2\cos{\Theta_B}^{(n)}}{\lambda}\left(\frac{\partial{\Theta_B}^{(n)}}{\partial(2\theta)}\Delta 2\theta + \frac{\partial{\Theta_B}^{(n)}}{\partial\beta}\Delta\beta\right) = \frac{\partial d^{*(n)}}{\partial\mathbb{Q}^*}\frac{\partial\mathbb{Q}^*}{\partial\mathbb{Q}}\Delta\mathbb{Q} \qquad (37),$$

where

$$\mathbb{Q} = \begin{cases} a_x, a_y, a_z, b_x, b_y, b_z, c_x, c_y, c_z & \text{in terms of the } \sigma \text{ tensor} \\ a, b, c, \alpha, \beta, \gamma & \text{in terms of lattice parameters'} \end{cases}$$

and $\Delta 2\theta$ and $\Delta\beta$ are the slit widths of the vertical tth-scan and horizontal beta-scan.. The uncertainties on the elements of the σ tensor and the lattice parameters of the nth layer can be assessed as below:

$$\Delta\mathbb{Q}^{(n)} = -\frac{-\frac{2\cos\Theta_B^{(n)}}{\lambda}\left(\frac{\partial\Theta_B^{(n)}}{\partial(2\theta)}\Delta 2\theta + \frac{\partial\Theta_B^{(n)}}{\partial\beta}\Delta\beta\right)}{\frac{\partial d^{*(n)}}{\partial\mathbb{Q}^*}\frac{\partial\mathbb{Q}^*}{\partial\mathbb{Q}}} \quad (38).$$

IX. Strain

The three unit lattice vectors, $\vec{a}$, $\vec{b}$ and $\vec{c}$, (also referred to in Supplement Ⅰ) may be rewritten in term of tensile strains, ε, and shear strains, τ, in a cubic system as a first-order approximation:

$$\vec{a} = (a_x \quad a_y \quad a_z) = a_0((1+\varepsilon_{xx}) \quad \tau_{yx} \quad \tau_{zx}),$$
$$\vec{b} = (b_x \quad b_y \quad b_z) = a_0(\tau_{xy} \quad (1+\varepsilon_{yy}) \quad \tau_{zy}),$$
$$\vec{c} = (c_x \quad c_y \quad c_z) = a_0(\tau_{xz} \quad \tau_{yz} \quad (1+\varepsilon_{zz})),$$

where the three symbols, ε_{xx}, ε_{yy} and ε_{zz}, are the x-, y- and z-direction tensile strains, and τ_{yx}, τ_{zx} are the y- and z-direction shear strains of a-axis. Similarly, τ_{xy}, τ_{zy} represent the x- and z-direction shear strains of b-axis, and τ_{xz}, τ_{yz} stand for the x-, y-direction shear strains of c-axis. The cubic lattice constant, a_0, is estimated by Vegard's law:

$$a_0 = xa_{si} + ya_{Ge} \quad (39),$$

where x and y are the composition of the Si and Ge, respectively, and the lattice constant of the cubic system for Si, a_{si}~5.43075 Å (see Ref. [4]) and Ge, a_{Ge}~5.6583 Å (Ref. [5]). From the equation (39) and Figure 1c, the lattice constant of the cubic system might be predicted in depth, and the result are shown in Supplementary Figure 4.

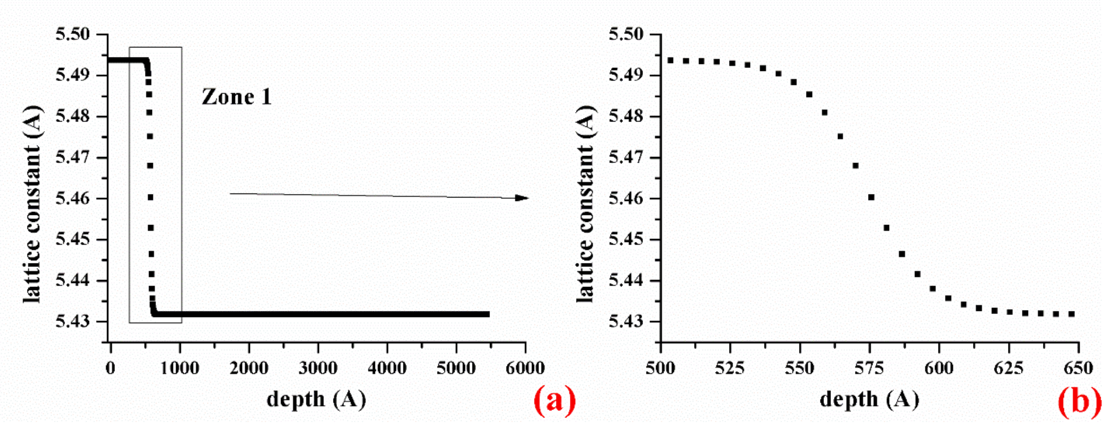

Figure 4| The prediction of lattice constant are estimated from Vegard's law and Figure 1c: (a) The lattice constant vs. depth, (b) the blow-up of Zone 1 in (a)

X. Double check the uniqueness of lattice parameter determination

For double-check the uniqueness of lattice parameter determination, we measured the intensity distributions for an additional three-beam BSD, (004)/(2 $\bar{4}$ 2) (see, Supplementary Figure 5), and perform the same simulations. The determined lattice parameters are within the error bars of the values given in Figure 4, the tth-scan is shown in Supplementary Figure 5a and the beta-scans at p1, p4 and at p2, p3 in Supplementary Figure 5b and 5c, respectively. The experimental conditions and changing rate c_0 are all the same as the previous ones.

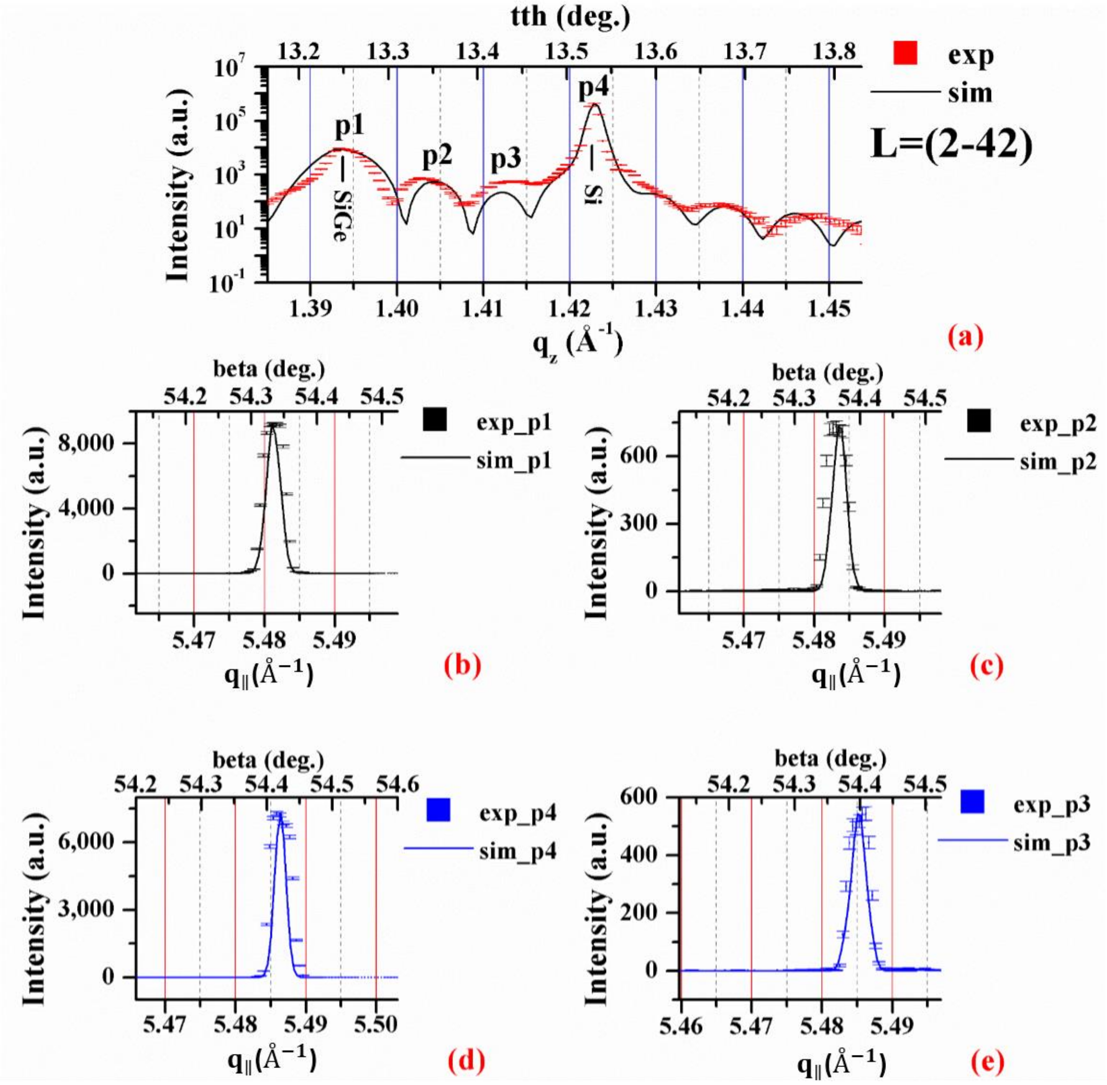

Figure 5 | The surface reflection intensity of (2$\bar{4}$2) (a) The calculation (curve in black) and measurement (square dot in red) of the tth-scan (or $\mathbf{q}_z$-scans), are carried out with the slit size, 10×0.1 mm (H× V), of the detector in the surface diffraction, 2$\bar{4}$2. The four beta-scans (or $\mathbf{q}_\parallel$-scans) are observed with the slit widths (0.1×0.1 mm, H× V) at the tth angle, **(b)** p1 (in black) **(c)** p2 (in black) **(d)** p4 (in blue) **(e)** p3 (in blue), respectively.

XI. **Figure**

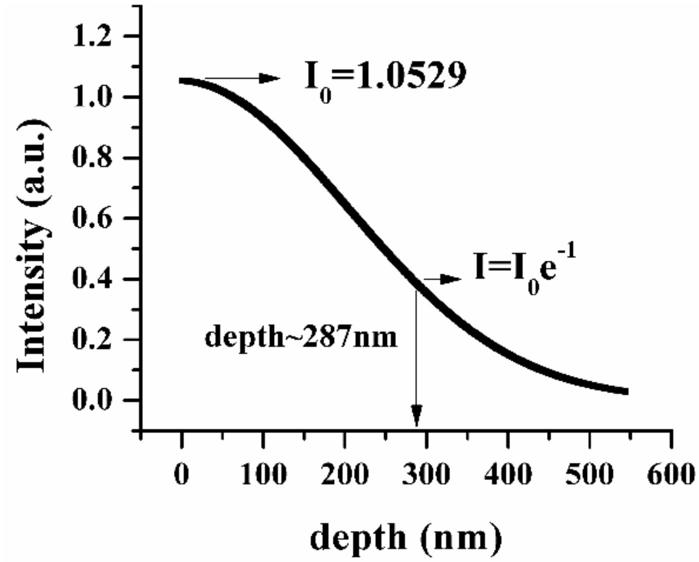

Figure 6 | Probing depth The diffracted intensity decays to e^{-1} at the depth (~287nm) .

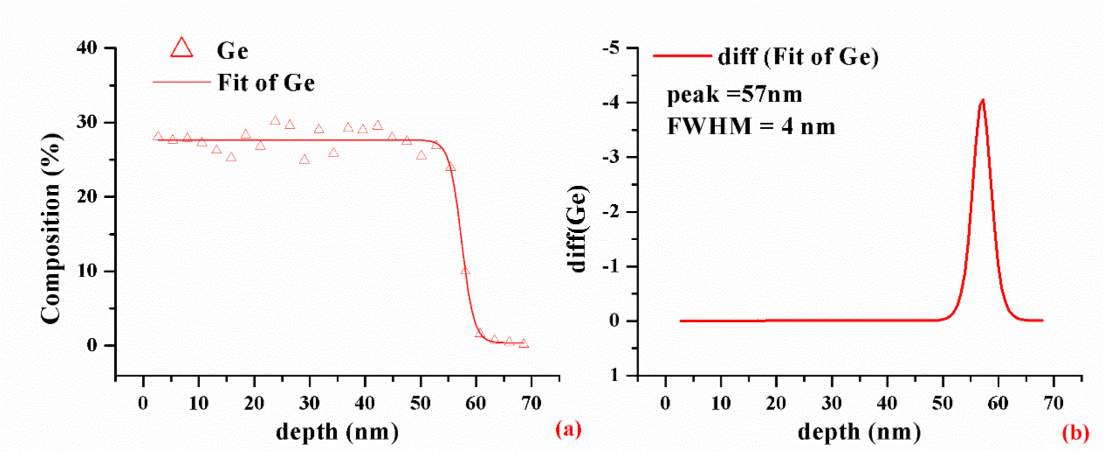

Figure 7 | (a) The EDS data (triangle) of Ge and its fitting curve (solid line) (b) The derivative of the fitting curve in (a) with respect to depth.

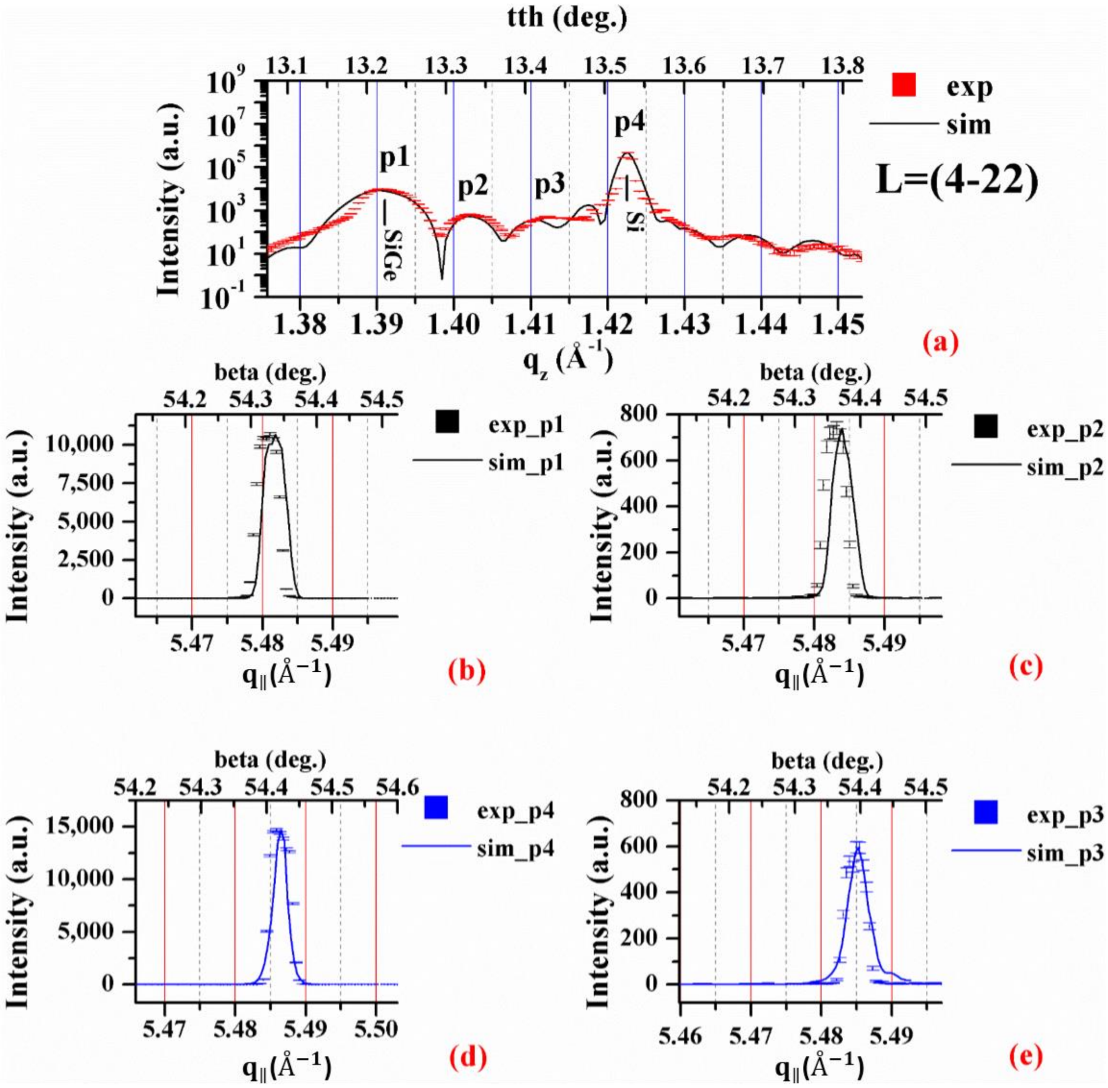

Figure 8 | Measured and simulated intensity distributions (a) the tth-scans of the reflection, (4-22), and the corresponding four beta-scans at the tth angles: **(b)** p1, **(c)** p2, **(d)** p4 and **(e)** p3 are shown with the two abscissas, beta angle (top) and $q_{\|}$ (bottom).

References

1. Stetsko, Y.P. and Chang , S.-L. An algorithm for solving multiple-wave dynamical X-ray diffraction equations. *Acta Cryst. A* **53,** 28-34 (1997).
2. Souvouov, A. *et al.* X-ray multiple diffraction from crystalline multilayers: Application to a 90° Bragg reflection. *Phys. Rev. B* **70,** 224109 (2004).
3. Reuter, M.G. and Hill, J.C. An efficient, block-by-block algorithm for inverting a block tridiagonal, nearly block Toeplitz matrix. *Comput. Sci. Discov.* **5,** 014009 (2012).
4. Bond, W.L. and Kaiser, W. Interstitial versus substitutional oxygen in silicon. *J. Phys. Chem. Solids* **16,** 44-45 (1960).
5. Dismukes, J.P., Ekstrom, L., and Paff, R.J. Lattice parameter and density in

germanium-silicon alloys. *J. Phys. Chem.* **68,** 3021-3027 (1964).
